# Bioactive Compounds from Mangrove-Associated Fungi as Leads Against ESKAPE Pathogens

**DOI:** 10.3390/life16081272

**Published:** 2026-07-31

**Authors:** Shivankar Agrawal, Laurent Dufossé, Sunil Kumar Deshmukh, Shilpa A. Verekar

**Affiliations:** 1 APC Microbiome Ireland, School of Microbiology, University College Cork, T12 YT20 Cork, Ireland; sagrawal@ucc.ie; 2 CHEMBIOPRO Laboratory, ESIROI Agro-Food, University of Reunion Island, 15 Avenue René Cassin-CS 92003, 97744 Saint-Denis, Reunion Island, France; 3 Research and Development, Greenvention Biotech Pvt. Ltd., Pune 411 041, India; 4 Central Quality Lab Operations, Asia Pacific, Middle East & Africa, Mumbai 400 099, India

**Keywords:** antibiotic resistance, antimicrobial, endophytic fungi, ESKAPE, marine natural product, priority pathogens, mangrove

## Abstract

The rapid emergence and dissemination of antimicrobial resistance (AMR) among bacterial pathogens, particularly the ESKAPE group (*Enterococcus faecium*, *Staphylococcus aureus*, *Klebsiella pneumoniae*, *Acinetobacter baumannii*, *Pseudomonas aeruginosa*, and *Enterobacter* spp.), represents one of the most pressing global public health challenges, contributing to increased morbidity, mortality, and healthcare costs. The limited development of new antibiotic classes over the past two decades has intensified the search for structurally novel antimicrobial agents and adjuvants capable of overcoming multidrug resistance. Natural products continue to serve as an invaluable source of anti-infective drug leads owing to their remarkable structural diversity and broad spectrum of biological activities. Mangrove ecosystems, located at the interface of terrestrial and marine environments, harbor highly diverse microbial communities, including fungi that have evolved under extreme environmental conditions and produce a wide range of unique secondary metabolites. Beyond their ecological significance, mangrove-associated fungi have emerged as prolific producers of bioactive compounds with promising antibacterial activity against multidrug-resistant pathogens. This review comprehensively summarizes recent advances (2018–2026) in the discovery of antibacterial metabolites from mangrove-associated fungi active against ESKAPE pathogens, which discusses their structural diversity, reported antimicrobial activities, and emerging strategies for accelerating natural product discovery, including genome mining, metabolomics, OSMAC, adaptive laboratory evolution, and artificial intelligence-assisted approaches. A total of 139 chemically distinct metabolites (Compounds **1**–**139**) isolated from mangrove-associated fungi are critically reviewed, highlighting their potential as promising leads for the development of next-generation antimicrobial agents against ESKAPE pathogens.

## 1. Introduction

Antimicrobial resistance (AMR) has emerged as one of the most pressing global public health challenges, threatening the effectiveness of current antibiotic therapies and significantly increasing morbidity, mortality, and healthcare costs. Recent estimates indicate that bacterial AMR was associated with approximately 4.95 million deaths globally in 2019, including 1.27 million deaths directly attributable to resistant bacterial infections, with projections suggesting that this burden will continue to escalate if new therapeutic strategies are not developed [1,2]. Among resistant bacteria, the ESKAPE pathogens- *Enterococcus faecium*, *Staphylococcus aureus*, *Klebsiella pneumoniae*, *Acinetobacter baumannii*, *Pseudomonas aeruginosa*, and *Enterobacter* spp.- are recognized as priority pathogens because of their remarkable ability to evade multiple classes of antibiotics through diverse resistance mechanisms, including biofilm formation, enzymatic drug inactivation, target modification, and active efflux systems [3,4]. Their increasing prevalence in both healthcare and community settings has created an urgent need to discover antibiotics with novel chemical scaffolds and mechanisms of action.

Natural products have historically served as the cornerstone of antibacterial drug discovery, with microorganisms contributing the majority of clinically used antibiotics. However, the rediscovery of known compounds from conventional terrestrial microorganisms has highlighted the necessity of exploring under investigated ecological niches for structurally diverse and biologically active metabolites [5,6]. Marine ecosystems, particularly mangrove habitats, represent one such promising but relatively underexplored source of microbial biodiversity. Mangrove-associated fungi inhabit dynamic environments characterized by fluctuating salinity, tidal inundation, nutrient limitation, and intense microbial competition, driving the evolution of unique biosynthetic pathways and chemically diverse secondary metabolites with potent biological activities [7,8].

In recent years, mangrove-associated fungi have emerged as prolific producers of structurally diverse natural products, including polyketides, alkaloids, terpenoids, peptides, xanthones, anthraquinones, and meroterpenoids, many of which exhibit potent antibacterial activity against multidrug-resistant pathogens, including members of the ESKAPE group [9]. Advances in genome mining, metabolomics, OSMAC (One Strain Many Compounds) strategies, co-cultivation, and epigenetic modulation have further accelerated the discovery of previously cryptic fungal metabolites with promising pharmaceutical potential [10,11,12].

Accordingly, this review comprehensively summarizes antibacterial metabolites isolated from mangrove-associated fungi reported between 2018 and 2026, with particular emphasis on their activity against ESKAPE pathogens. Scientific databases such as Scopus, Web of Science, PubMed and Google Scholar were used for literature search using keywords “Mangrove Fungi” “Mangrove Associated Fungi” “Fungal metabolites”. We discuss the ecological diversity of mangrove-associated fungi, the structural classes and mechanisms of action of their antibacterial metabolites, emerging trends in natural product discovery, and current challenges and future perspectives for translating these promising compounds into clinically useful antibacterial agents.

## 2. Resistance in ESKAPE Pathogens

**Figure 1:**
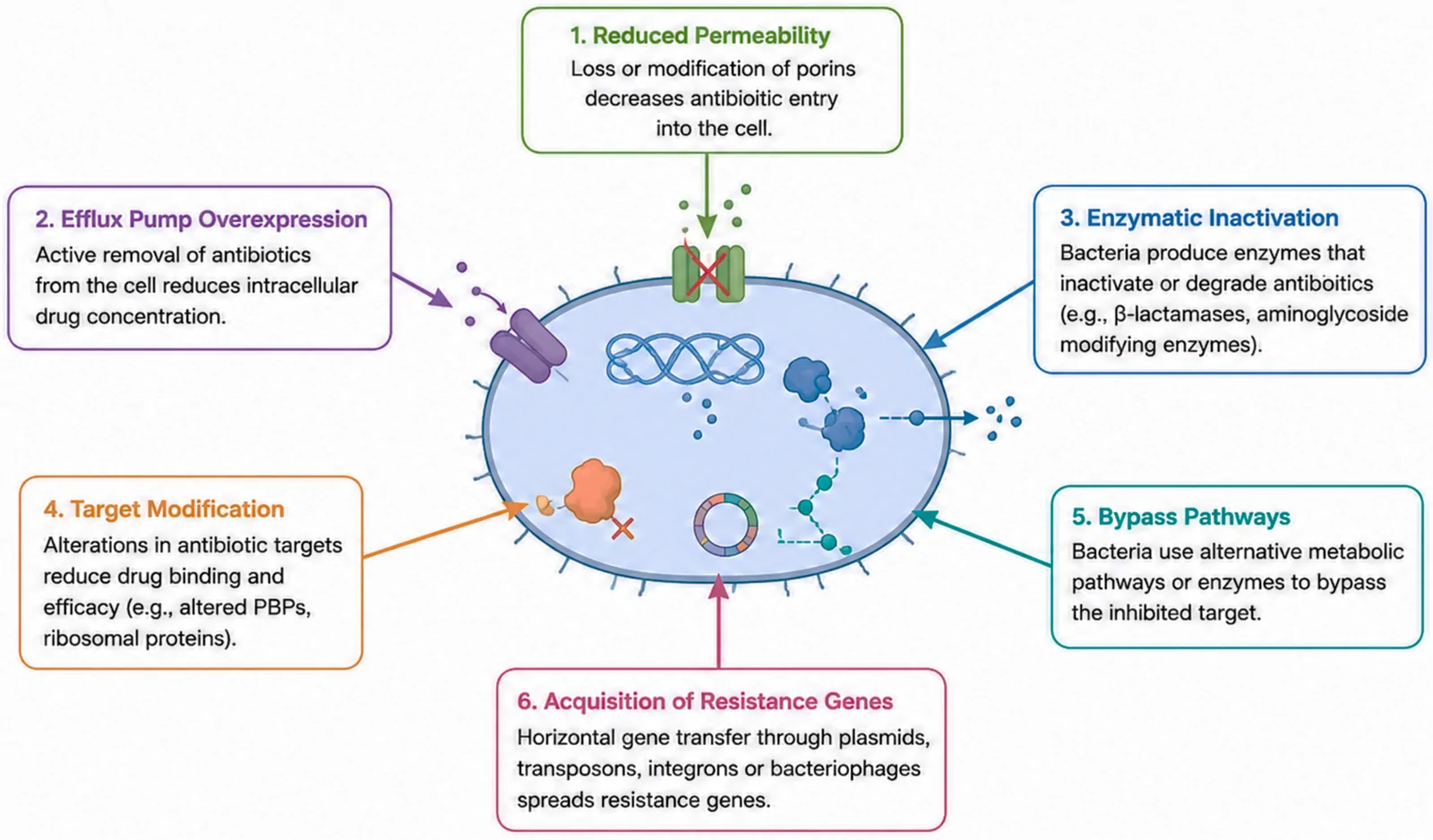
Mechanisms facilitating antimicrobial resistance in ESKAPE pathogens.

The proliferation of antibiotic resistance within bacterial communities has led to a concerning decline in the effectiveness of antimicrobial treatments. Over recent decades, the escalation of antibiotic resistance has raised alarms among experts, who caution that humanity is approaching a post-antibiotic era [13,14]. ESKAPE bacteria, including *Enterococcus faecium*, *Staphylococcus aureus*, *Klebsiella pneumoniae*, *Acinetobacter baumannii*, *Pseudomonas aeruginosa*, and *Enterobacter* spp., are increasingly linked to antibiotic resistance, posing a significant threat to public health (Figure 1) [15,16,17]. *Enterococcus faecium* is an opportunistic pathogen that commonly causes urinary tract infections (UTIs), bloodstream infections (bacteremia), infective endocarditis, intra-abdominal infections, surgical site infections, and neonatal sepsis, with symptoms including fever, dysuria, abdominal pain, fatigue, and signs of systemic inflammation [18]. *Staphylococcus aureus* causes skin and soft tissue infections such as boils, abscesses, cellulitis, and impetigo, as well as pneumonia, osteomyelitis, septic arthritis, bacteremia, endocarditis, and toxic shock syndrome. Clinical manifestations range from localized pain, swelling, erythema, and purulent lesions to high fever, respiratory distress, hypotension, and multi-organ failure [19]. *Klebsiella pneumoniae* primarily causes community- and hospital-acquired pneumonia, UTIs, liver abscesses, bacteremia, meningitis, and septic shock, presenting with fever, productive cough, dyspnea, chest pain, dysuria, flank pain, and systemic sepsis [20]. *Acinetobacter baumannii* is predominantly associated with ventilator-associated pneumonia, bloodstream infections, wound and burn infections, meningitis, catheter-related infections, and UTIs, especially in critically ill patients. Symptoms include persistent fever, purulent wound discharge, respiratory distress, altered mental status, and septic shock [21]. *Pseudomonas aeruginosa* is a highly versatile pathogen responsible for pneumonia, chronic lung infections in individuals with cystic fibrosis, burn and wound infections, UTIs, keratitis, otitis externa, bacteremia, and endocarditis. Clinical features include fever, productive cough, dyspnea, purulent wound exudate, severe pain, urinary discomfort, and bloodstream infections that may progress to sepsis [22]. *Enterobacter* species, including *Enterobacter cloacae* complex, are increasingly recognized as causes of healthcare-associated pneumonia, UTIs, bloodstream infections, intra-abdominal infections, surgical site infections, neonatal sepsis, and meningitis. Patients typically present with fever, chills, dysuria, abdominal pain, respiratory symptoms, and signs of systemic infection. The increasing prevalence of multidrug-resistant strains within all ESKAPE pathogens significantly complicates treatment, leading to prolonged hospitalization, increased morbidity and mortality, and greater economic losses in healthcare [23].

These bacteria carry genes encoding resistance to commonly used classes of antibiotics such as vancomycin, methicillin, broad-spectrum β-lactams, carbapenems, fluoroquinolones, and aminoglycosides [24]. In 2020, the European Centre for Disease Prevention and Control (ECDC), through its European Antimicrobial Resistance Surveillance Network (EARS-Net), published a report on antimicrobial resistance among ESKAPE pathogens in the European Union [25]. Of particular concern is the rise in the percentage of vancomycin-resistant enterococci (VRE) harboring the vanA gene [26]. *Staphylococcus aureus*, the second most worrying pathogen, as it does not have a set number; rather, the bacteria carry hundreds of unique, acquired resistance genes across various mobile genetic elements. However common resistance genotypes found in *S. aureus* include; methicillin-resistant *S. aureus* (MRSA) with the mecA, mecC and blaZ gene and vancomycin-resistant *S. aureus* (VRSA) with the vanA gene or vanB gene operons [27]. Macrolide and Lincosamide resistance genes like ermA, ermB, and ermC. Tetracycline Resistance: Frequently mediated by genes such as tetK, tetM, and tet38 [28]. *Klebsiella pneumoniae* has also developed various β-lactamase enzymes, including blaNDM and blaKPC genes, enabling it to resist penicillins, cephalosporins, and carbapenems, which are considered last-resort drugs [29]. *Acinetobacter baumannii*, an emerging human pathogen, can easily acquire and transfer antibiotic resistance genes (ARGs) to other bacteria. It carries Ambler class B and D genes that confer resistance to carbapenems, posing a significant challenge in the medical field [30]. The ESKAPE group also includes *Pseudomonas aeruginosa* and *Enterobacter* spp., both of which are major contributors to multidrug-resistant healthcare-associated infections but employ distinct resistance mechanisms. *Pseudomonas aeruginosa* exhibits high levels of intrinsic and acquired antimicrobial resistance through multiple mechanisms, including the overexpression of multidrug efflux pumps (e.g., MexAB-OprM, MexXY), reduced outer membrane permeability caused by loss or modification of the OprD porin, biofilm formation, target-site mutations, and the production of carbapenemases, particularly metallo-β-lactamases such as IMP, VIM, and NDM [31]. In contrast, *Enterobacter* spp. commonly develops resistance through the production of extended-spectrum β-lactamases (ESBLs), including **bla**_CTX-M_, **bla**_SHV_, and **bla**_TEM_, as well as AmpC β-lactamase overexpression and carbapenemases, which confer resistance to a broad range of β-lactam antibiotics [32,33]. In 2017, WHO published a priority pathogen list (as described by Tacconelli et al., 2018) of antibiotic-resistant priority pathogens that threaten human health, with all ESKAPE bacteria included on that list [34]. Based on these considerations, it has been determined that our review will encompass mangrove-associated fungal metabolites with inhibitory effects on bacterial growth. Consequently, our focus will be directed towards an examination of their impact on the extended ESKAPE pathogenic bacteria.

## 3. The Mangrove -Associated Fungal Community Throughout History

Marine fungal species remain significantly less studied than their land-based counterparts, often receiving minimal scientific attention [35,36]. By 2011, researchers had documented just 537 species of fungi that live exclusively in ocean and brackish water environments [37]. However, current understanding suggests this figure vastly underrepresents the true diversity of marine fungal life, with cautious projections indicating that scientists may eventually identify more than 10,000 additional marine fungal species [37,38,39].

The earliest scientific documentation of fungi living in association with mangrove trees appeared in 1955, when Cribb and Cribb observed fungal growth on mangrove root systems [40]. This initial work was expanded by Kohlmeyer in 1969, who catalogued observable fungal species across various tropical mangrove populations [41]. Contemporary research on mangrove fungi has predominantly concentrated on either cataloguing previously unidentified species or investigating compounds produced by these fungi [9,42]. Studies employing DNA-based methods to analyze fungal communities have typically examined isolated plant components such as individual leaves rather than investigating fungi across entire mangrove ecosystems and their surrounding habitats [43,44].

Salt-tolerant plant communities known as mangroves thrive in coastal zones where tropical and subtropical rivers meet the ocean. These ecosystems feature partially flooded woody vegetation and, despite covering merely 1% of all tropical forests globally, deliver critical ecological services to shoreline areas across warm climate regions. These transitional zones between terrestrial and marine environments support diverse biological communities, including various plant species alongside populations of mammals, birds, aquatic organisms, and invertebrates [45].

The challenging conditions where mangrove vegetation flourishes including high salinity, fluctuating tidal inundation, and oxygen-poor sediments paradoxically create favorable niches for diverse microbial communities. These environments are enriched with organic carbon and nitrogen substrates derived from plant litter and tidal inputs, while persistent moisture supports active microbial metabolism and nutrient cycling. However, climatic conditions vary considerably across mangrove regions; although tropical mangroves may experience mean annual temperatures close to 30 °C, subtropical mangrove systems, such as those extensively studied along the Chinese coastline, often occur under cooler conditions with average annual temperatures around 20–25 °C. Environmental parameters, particularly temperature and salinity, strongly influence microbial community composition and functional potential in mangrove sediments [46]. These environments harbor distinctive communities of fungi, bacteria, and other microscopic life forms [47]. Fungi inhabiting mangrove systems fall into two categories: those living within plant tissues (endophytic species) and free-living forms. Both groups have attracted scientific interest due to their capacity to synthesize biologically active molecules. Endophytic fungi colonize mangrove plants extensively and contribute important physiological benefits, helping their hosts withstand environmental and biological challenges. These organisms represent valuable sources of novel compounds with potential applications and industrially relevant enzymes. Research has identified numerous fungal species from mangrove habitats capable of producing bioactive substances, including multiple species from the genera *Penicillium*, *Aspergillus*, *Talaromyces*, and *Diaporthe*, among others [48]. Each species generates distinct compounds with varied biological properties, antimicrobial activities were interpreted according to the following MIC scale: strong (≤10 µg/mL), moderate (10–100 µg/mL), weak (100–500 µg/mL), and inactive (>500 µg/mL), as summarized in Supplementary Table S1.

### 3.1. Anti-ESKAPE Metabolites from Penicillium spp.

**Figure 2:**
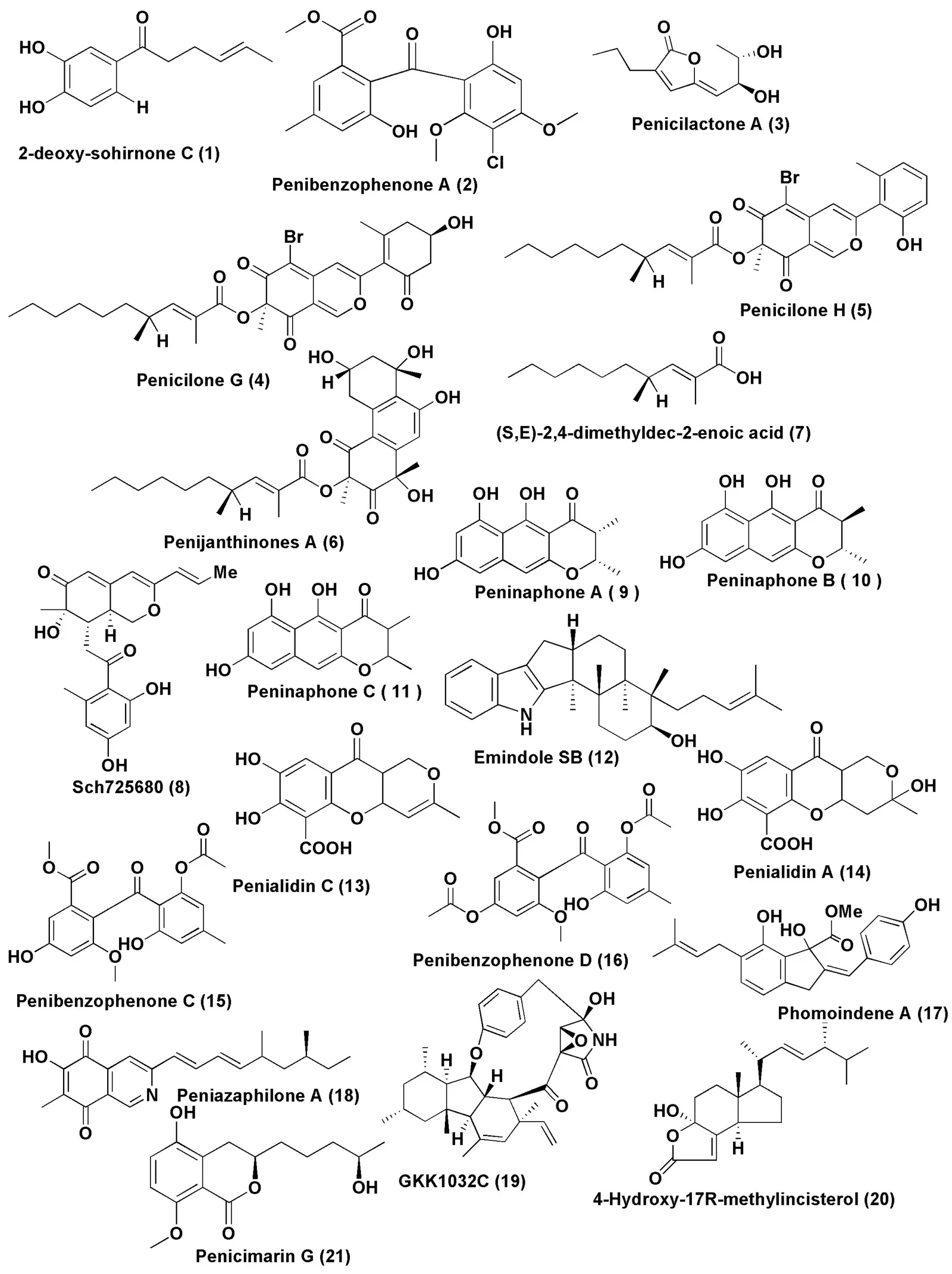

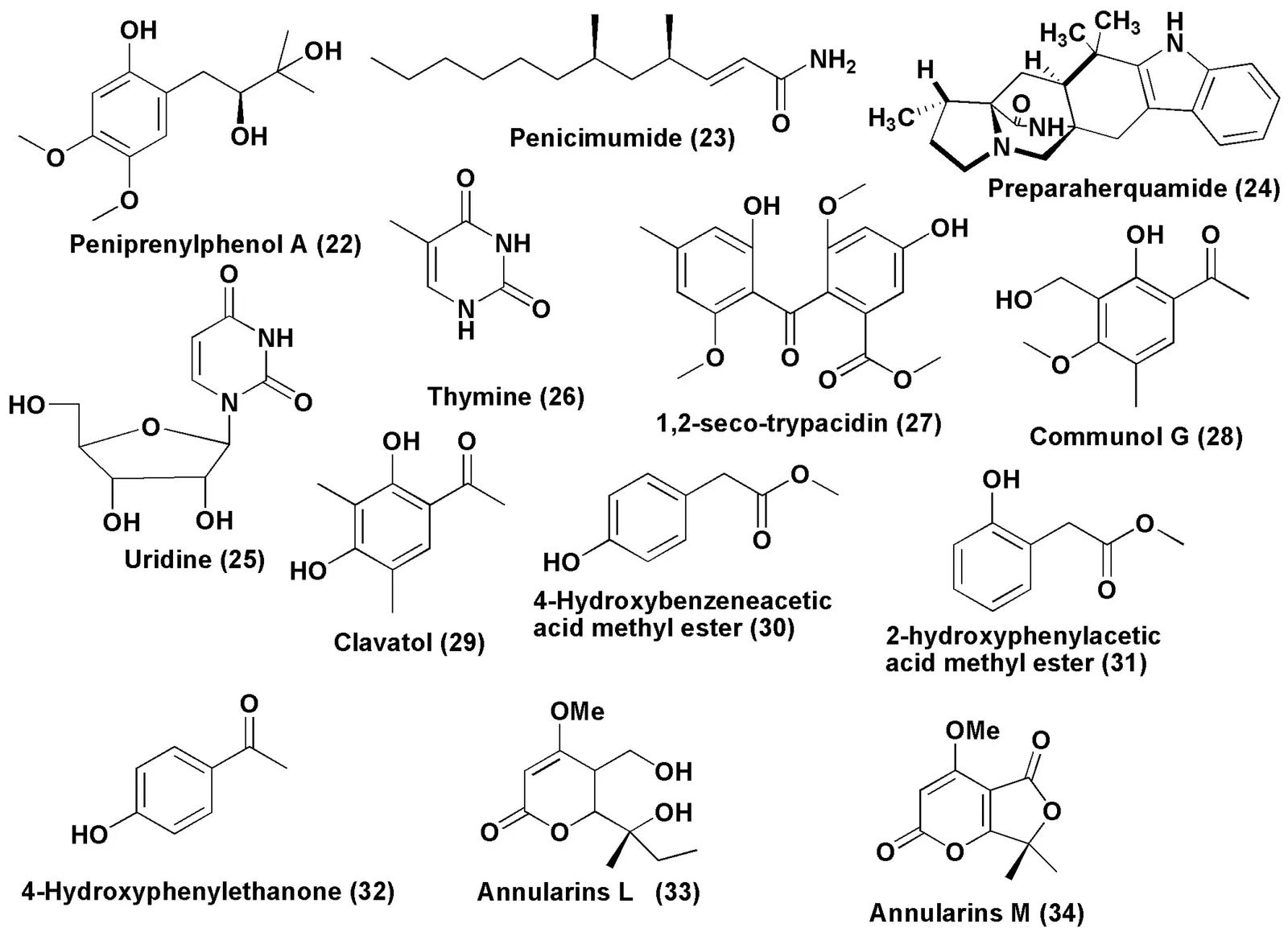
Anti-ESKAPE Metabolites from Mangrove-Associated *Penicillium* spp.

A novel sorbicillin derivative, 2-deoxy-sohirnone C (**1**) (Figure 2), was isolated from the Chinese mangrove-derived endophytic fungus *Penicillium* sp. GD6, which had been obtained from the stem bark of *Bruguiera gymnorrhiza* collected in Zhanjiang, China. Compound (**1**) exhibited moderate antibacterial activity against methicillin-resistant *Staphylococcus aureus* (MRSA), with a minimum inhibitory concentration (MIC) value of 80 μg/mL [49].

A new benzophenone derivative, penibenzophenone A (**2**) (Figure 2), was obtained from *Penicillium citrinum* HL-5126, isolated from the mangrove plant *Bruguiera sexangula* var. *rhynchopetala* collected from the South China Sea. Compound (**2**) demonstrated antibacterial activity against *S. aureus* with an MIC value of 20 μg/mL [50].

The new lactone metabolite penicilactone A (**3**) (Figure 2) was purified from *Penicillium* sp. TGM112, which was isolated from *B. sexangula* var. *rhynchopetala* collected in the South China Sea. Compound **3** displayed antibacterial activity against *S. aureus* with an MIC value of 6.25 μg/mL, while ciprofloxacin, used as the positive control, exhibited an MIC value of 0.39 μg/mL [51].

From *Penicillium janthinellum* HK1-6, isolated from mangrove rhizosphere soil collected in the Dongzhaigang Mangrove Natural Reserve, Hainan Island, two brominated azaphilones, penicilones G and H (**4–5**), two tricyclic polyketides, penijanthinones A (**6**), and an ester hydrolysis product, (S,E)-2,4-dimethyldec-2-enoic acid (**7**) (Figure 2), were characterized following NaBr supplementation. Compounds **4** and **5** exhibited moderate to strong antibacterial activity against several Gram-positive bacteria, including *S. aureus* strains (ATCC 43300, 33591, 29213, and 25923), and *E. faecium* ATCC 35667, with MIC values ranging from 3.13 to 25 μg/mL. Compound **6** also showed significant inhibitory effects against the same bacterial strains, with MIC values within the same range. Compounds **5** and **7** displayed potent activity against methicillin-resistant *S. aureus* ATCC 33591, each with an MIC value of 3.13 μg/mL, which was within a 2-fold range of vancomycin (MIC 1.56 μg/mL). Compound **7** further demonstrated moderate antibacterial effects against *S. aureus* ATCC 43300 and ATCC 33591, both with MIC values of 25 μg/mL [52].

The known metabolite Sch725680 (**8**) (Figure 2) was isolated from the mangrove sediment-derived fungus *Penicillium pinophilum* SCAU037, obtained from the roots of *Rhizophora stylosa* collected on Techeng Isle, China. This compound showed antibacterial activity against *S. aureus*, with an IC_50_ value of 1.01 µg/mL [53].

Three novel monomeric naphtho-γ-pyrones, peninaphones A–C (**9–11**) (Figure 2), were isolated from *Penicillium* sp. HK1-22, derived from mangrove rhizosphere soil collected in the Dongzhaigang Mangrove Natural Reserve, Hainan Island. These compounds displayed antibacterial activity against several *S. aureus* strains (ATCC 43300, 33591, 29213, and 25923), with MIC values ranging from 12.5 to 50 μg/mL. Vancomycin, used as the positive control, exhibited MIC values of 1.56, 0.39, 1.56, and 0.78 μg/mL, respectively [54].

In another study involving *Penicillium* sp. HK1-22 from the same mangrove rhizosphere soil, two known naphtho-γ-pyrones, peninaphone A (**9**) and peninaphone B (**10**) (Figure 2), were obtained. Both compounds exhibited weak to strong antibacterial activity against Gram-positive bacteria, including methicillin-resistant *S. aureus* (ATCC 33591 and 43300), sensitive *S. aureus* (ATCC 29213 and 25923), and *E. faecium* ATCC 35667. Antibacterial activity was evaluated using the filter disc method, producing inhibition zones ranging from 10.4 to 21.0 mm [55].

The indole diterpene emindole SB (**12**) and two polyketides, penialidin C (**13**) and penialidin A (**14**) (Figure 2), were isolated from *Penicillium javanicum* HK1-23 collected from mangrove rhizosphere soil in the Dongzhaigang Mangrove Natural Reserve, Hainan Island. Compound **13** demonstrated potent antibacterial activity against *S. aureus* strains ATCC 43300, 33591, 25923, and 29213, with MIC values ranging from 0.78 to 25 μg/mL. Particularly, it exhibited strong activity against MRSA ATCC 43300 with an MIC value of 0.78 μg/mL. Compound **14** also displayed antibacterial effects against *S. aureus* ATCC 43300 and ATCC 29213, with MIC values of 3.13 and 12.5 μg/mL, respectively. Emindole SB (**12**) showed selective activity toward *S. aureus* ATCC 33591 with an MIC value of 6.25 μg/mL. Vancomycin, used as the reference antibiotic, showed MIC values of 0.78, 6.25, 3.13, and 0.78 μg/mL against the tested strains, respectively [56].

Two new benzophenone derivatives, penibenzophenone C (**15**) and penibenzophenone D (**16**) (Figure 2), were isolated from a *Penicillium* species associated with the mangrove plant *Acanthus ilicifolius* L. collected from the South China Sea. Both compounds showed antibacterial activity against MRSA, with MIC values of 3.12 and 6.25 μg/mL, respectively. Ciprofloxacin served as the positive control with an MIC value of 1.56 μg/mL [57].

The compound 1,7-dihydroxy-2(Z)-(4-hydroxybenzylidene)-6-(3-methylbut-2-enyl)-indane-1-carboxylic acid methyl ester (**17**) (Phomoindene A) (Figure 2) was isolated from *Penicillium verruculosum* TGM14 associated with *Xylocarpus granatum* collected from the South China Sea. This metabolite exhibited antibacterial activity against *S. aureus* with an MIC value of 50 μg/mL [58].

Co-cultivation of the mangrove-derived fungi *Penicillium sclerotiorum* THSH-4 and *Penicillium sclerotiorum* ZJHJJ-18, both isolated from *Aegiceras corniculatum* collected from the Huizhou East Mangrove National Nature Reserve in Guangdong Province, China, resulted in the production of a new azaphilone derivative, peniazaphilone A (**18**) (Figure 2). This compound exhibited moderate antibacterial activity against *Pseudomonas aeruginosa* and MRSA, with MIC values of 12.5 μmol/L [59].

Two known metabolites, GKK1032C (**19**) and 4-hydroxy-17R-methylincisterol (**20**) (Figure 2), were isolated from the mangrove endophytic fungus *Penicillium steckii* SCSIO 41025 associated with *Avicennia marina* collected from Zhanjiang, Guangdong Province, China. These compounds exhibited antibacterial activity against *S. aureus*, with MIC values of 3.9 and 7.8 μg/mL, respectively, whereas gentamicin used as the positive control showed an MIC value of 0.5 μg/mL [60].

The known dihydroisocoumarin penicimarin G (**21**) (Figure 2) was isolated from *Penicillium verruculosum* associated with the mangrove plant *Xylocarpus granatum* Koenig collected from the South China Sea. Compound **21** displayed antibacterial activity against *S. aureus* with an MIC value of 25 μg/mL [61].

A novel tetrasubstituted benzene derivative, peniprenylphenol A (**22**), together with several known compounds including penicimumide (**23**), preparaherquamide (**24**), uridine (**25**), thymine (**26**), 1,2-seco-trypacidin (**27**), communol G (**28**), clavatol (**29**), 4-hydroxybenzeneacetic acid methyl ester (**30**), 2-hydroxyphenylacetic acid methyl ester (**31**), and 4-hydroxyphenylethanone (**32**) (Figure 2), were isolated from *Penicillium chrysogenum* ZZ1151 obtained from mangrove sediment collected in the tidal flats of Pangkep District, South Sulawesi Province, Indonesia. Peniprenylphenol A (**22**) showed antibacterial activity against MRSA with an MIC value of 6 μg/mL. Compounds **24**, **25**, **27**, and **30** also demonstrated activity against MRSA with MIC values ranging from 3 to 25 μg/mL. Compounds **26** and **29** were active against MRSA with MIC values between 13 and 25 μg/mL, while compound **31** exhibited an MIC value of 13 μg/mL. In addition, compounds **28** and **32** showed antibacterial activity against MRSA with MIC values of 25 μg/mL [62].

Two new α-pyrone derivatives, annularins L and M (**33–34**) (Figure 2), were isolated from *Penicillium herquei* MA-370, a fungus obtained from the rhizospheric soil of the mangrove plant *Rhizophora mucronata* collected on Hainan Island, China. These compounds displayed antibacterial activity against *Pseudomonas aeruginosa* QDIO-4 with MIC values of 64 μg/mL. Chloramphenicol, used as the positive control, exhibited an MIC value of 2 μg/mL against the same strain [63].

### 3.2. Anti-ESKAPE Metabolites from Aspergillus spp.

**Figure 3:**
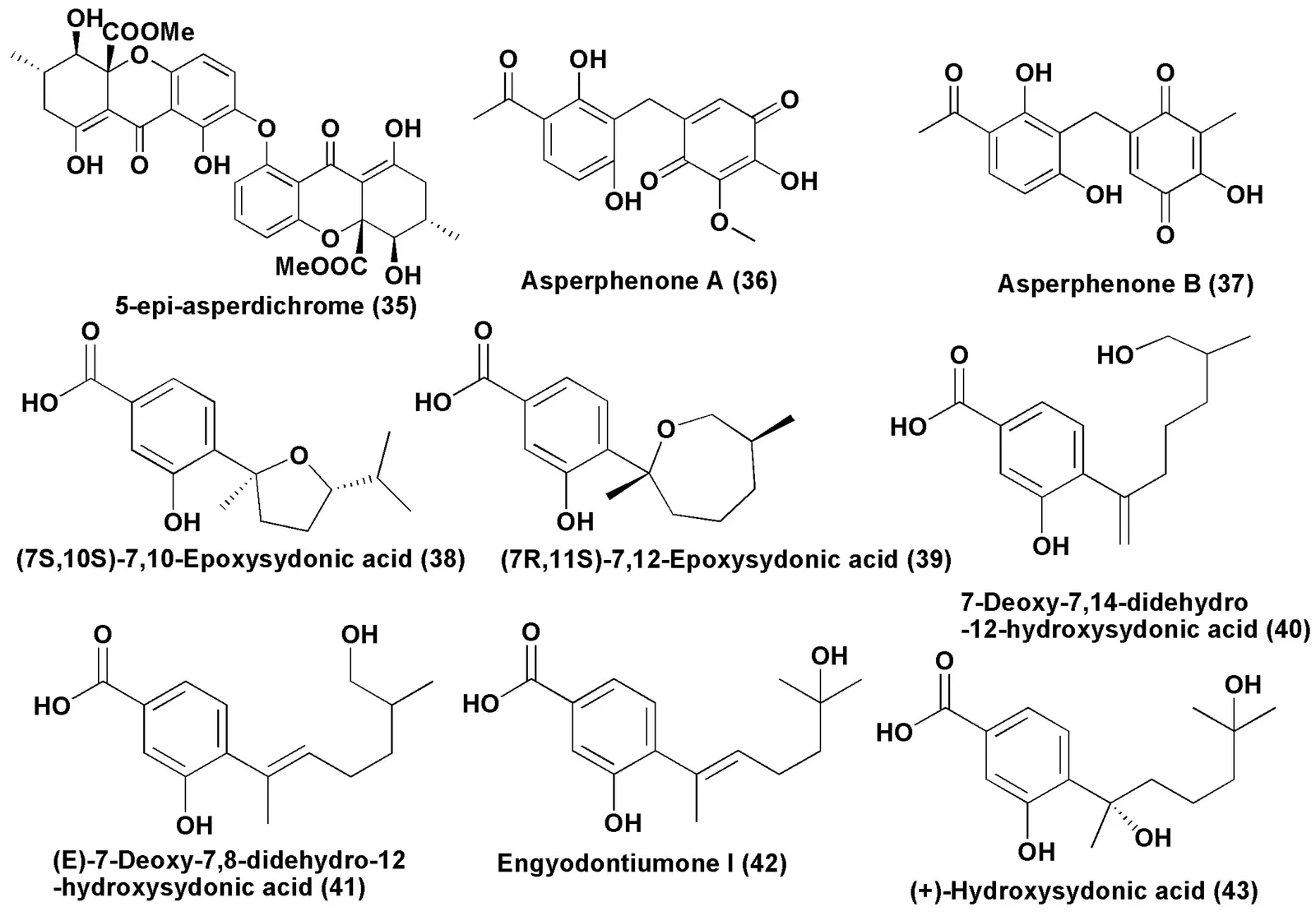

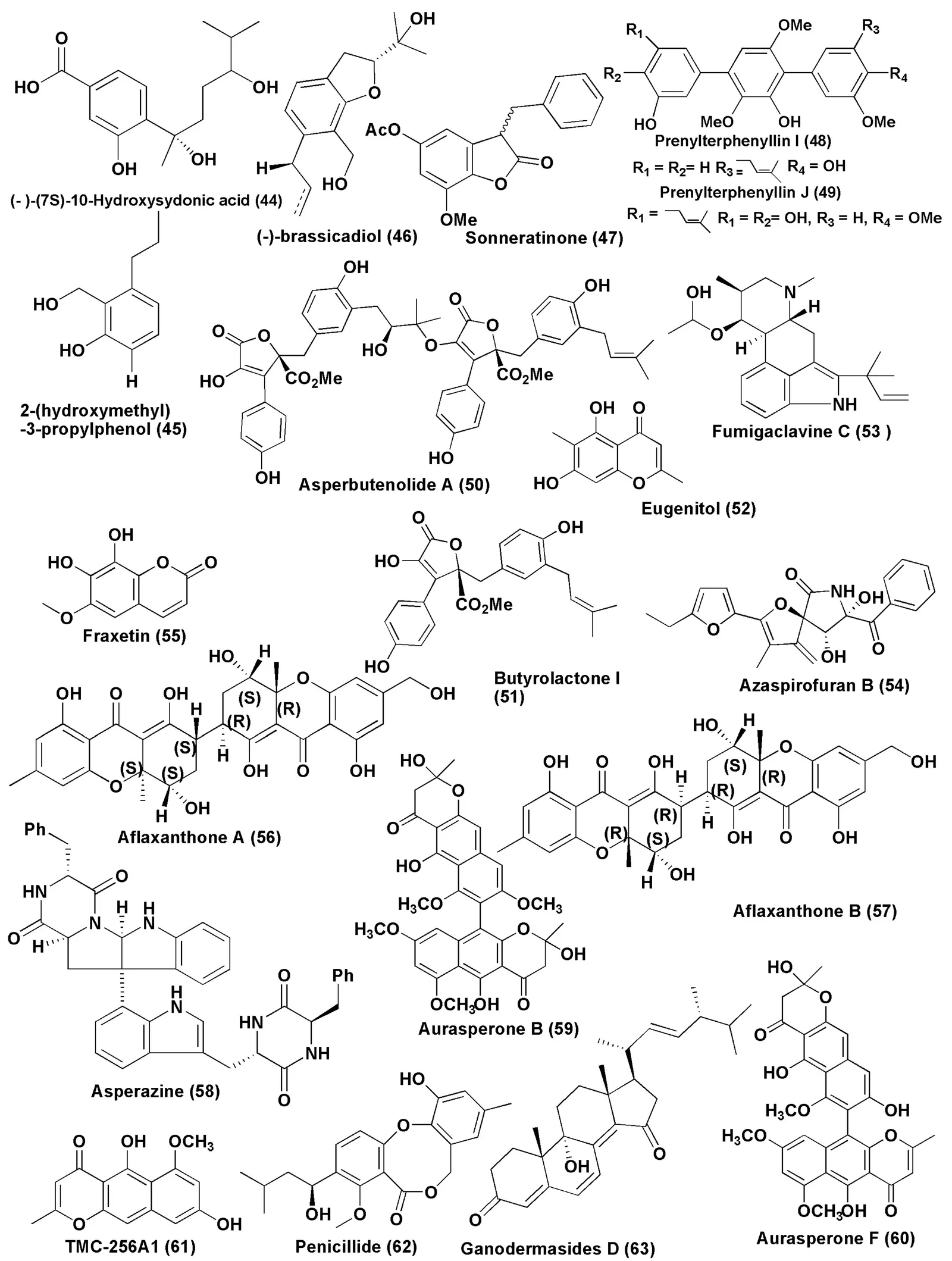
Anti-ESKAPE Metabolites from Mangrove-Associated *Aspergillus* spp.

A new tetrahydroxanthone dimer, 5-epi-asperdichrome (**35**) (Figure 3), was isolated from the mangrove-derived fungus *Aspergillus versicolor* HDN1009, which had been obtained from mangrove-associated soil collected in Guangzhou, China. Compound (35) demonstrated mild antibacterial activity against *Pseudomonas aeruginosa* with a MIC value of 100 μM. In comparison, the positive control Ciprofloxacin showed considerably stronger antibacterial activity with a MIC value of 1.56 μM [64].

Two new phenone derivatives, asperphenone A-B (**36–37**) (Figure 3), were obtained from the mangrove-derived fungus *Aspergillus* sp. YHZ-1. Compounds (**36**) and (**37**) exhibited antibacterial effects against *Staphylococcus aureus* CMCC(B) 26003 with MIC value ranging from 32.0 to 64.0 μM. The reference antibiotic Ampicillin displayed antibacterial activity against *S. aureus* CMCC(B) 26003 with a MIC value of 4.0 μM [65].

Four new phenolic bisabolane sesquiterpenoids, namely (7S,10S)-7,10-epoxysydonic acid (**38**), (7R,11S)-7,12-epoxysydonic acid (**39**), 7-deoxy-7,14-didehydro-12-hydroxysydonic acid (**40**), and (E)-7-deoxy-7,8-didehydro-12-hydroxysydonic acid (**41**), together with the known analogues engyodontiumone I (**42**), (+)-hydroxysydonic acid (**43**), and (−)-(7S)-10-hydroxysydonic acid (**44**) (Figure 3), were isolated from the endophytic fungus *Aspergillus* sp. xy02 associated with the mangrove plant *Xylocarpus moluccensis* collected in Trang Province, Thailand. Compounds (**38–44**) showed moderate inhibitory effects against *S. aureus* ATCC 25923, with IC50 values of 32.2, 36.0, 41.9, 31.5, 33.4, 34.0, and 36.3 μM, (respectively. By comparison, the positive controls Penicillin and Ceftriaxone demonstrated IC_50_ values of 23.6 and 1.3 μM, respectively [66].

wo previously known compounds, 2-(hydroxymethyl)-3-propylphenol (**45**) and (−)-brassicadiol (**46**) (Figure 3), were isolated from *Aspergillus sp*. ZJ-68 obtained from *Kandelia candel* collected in the Zhanjiang Mangrove Nature Reserve, Guangdong Province, China. Both compounds exhibited antibacterial activity against *S. aureus*, with MIC values ranging from 4.15 to 12.5 μg/mL. The positive control Ciprofloxacin showed stronger activity, with a MIC value of 1.25 μg/mL [67].

A new antimicrobial benzofuranone derivative, sonneratinone (**47**) (Figure 3), was isolated from the endophytic fungus *Aspergillus niger* associated with *Sonneratia apetala* collected from the Sundarbans. Sonneratinone (**47**) demonstrated antimicrobial activity against *S. aureus* with a MIC value of 2.5 × 10^−1^ mg/mL and against *P. aeruginosa* (NCTC 12903) with a MIC value of 5 × 10^−1^ mg/mL. In contrast, the reference antibiotic Ciprofloxacin exhibited significantly lower MIC values of 9.8 × 10^−4^ mg/mL against *S. aureus* and 1.2 × 10^−4^ mg/mL against *P. aeruginosa* [68].

Two previously undescribed prenylated *p*-terphenyl derivatives, prenylterphenyllins I (**48**) and J (**49**) (Figure 3), were isolated from *Aspergillus candidus* LDJ-5 derived from the roots of *Rhizophora apiculata* collected in Sanya Bailu Park, Hainan Province, China. Compound (**48**) exhibited antibacterial activity against *P. aeruginosa* with an MIC value of 42 μg/mL, whereas compound (**49**) displayed a MIC value of 90 μg/mL. The positive control Ciprofloxacin showed substantially greater activity with a MIC value of 0.52 μg/mL [69].

A new dimeric metabolite, asperbutenolide A (**50**), together with the known analogue butyrolactone I (**51**) (Figure 3), was isolated from *Aspergillus terreus* SCAU011 recovered from the rhizosphere soil of the mangrove plant *Rhizophora stylosa*. Compound (**50**) displayed moderate growth inhibitory activity against *S. aureus* with IC_50_ values of 1.35 μM. The positive control Penicillin exhibited an IC_50_ value of 0.130 μM against *S. aureus* [70].

The polyketide derivative eugenitol (**52**) (Figure 3) was isolated from *Aspergillus* sp. SCSIO41407 obtained from mangrove sediment collected near the Hongsha River estuary in the South China Sea, Sanya City, Hainan Island. Compound (**52**) exhibited weak antibacterial activity against methicillin-resistant *Staphylococcus aureus* (MRSA), with a MIC value of 485.4 μM [71].

Compounds fumigaclavine C (**53**), azaspirofuran B (**54**), and fraxetin (**55**) (Figure 3) were isolated from *Aspergillus fumigatus* associated with *Ceriops decandra* collected from the Sundarbans. The ethyl acetate extract containing these compounds demonstrated antibacterial activity against *S. aureus* and *P. aeruginosa*, with MIC values of 0.625 and 0.156 mg/mL, respectively [72].

Two new heterodimeric tetrahydroxanthones, aflaxanthones A (**56**) and B (**57**) (Figure 3), characterized by an unusual 7,7′-sp3–sp3 linkage, were isolated from the mangrove endophytic fungus *Aspergillus flavus* QQYZ obtained from *Kandelia candel* collected in Huizhou, Guangdong Province, China. Compound (**56**) exhibited moderate inhibitory activity against MRSA with a MIC value of 12.5 μM. The positive control, Ampicillin displayed a MIC value of 0.39 μM against MRSA [73].

Compounds asperazine (**58**), aurasperone B (**59**), aurasperone F (**60**), TMC-256A1 (**61**), and penicillide (**62**) (Figure 3) were isolated from *Aspergillus* sp. IQ-548 recovered from mangrove soil, leaves, and roots of *Rhizophora mangle* and *Laguncularia racemosa* collected from lagoons in Oaxaca, Mexico. Compounds (**58–61**) inhibited the growth of multidrug-resistant *Acinetobacter baumannii* A564 with IC_50_ values of 6.9, 9.9, 8.3, and 7.4 μg/mL, respectively, whereas compound (**62**) exhibited 40% inhibition at 100 μg/mL. The positive control Gentamicin showed 20% inhibition at 100 μg/mL against multidrug-resistant *A. baumannii* A564 [74]. A known sterol analogue, ganodermasides D (**63**) (Figure 3), was isolated from the mangrove-derived fungus *Aspergillus terreus* GXIMD 03158 associated with the mangrove plant *Acanthus ilicifolius*. Compound (**63**) exhibited significant antibacterial activity against *S. aureus*, with a MIC value of 6.25 μg/mL [75].

### 3.3. Anti-ESKAPE Metabolites from Talaromyces spp.

**Figure 4:**
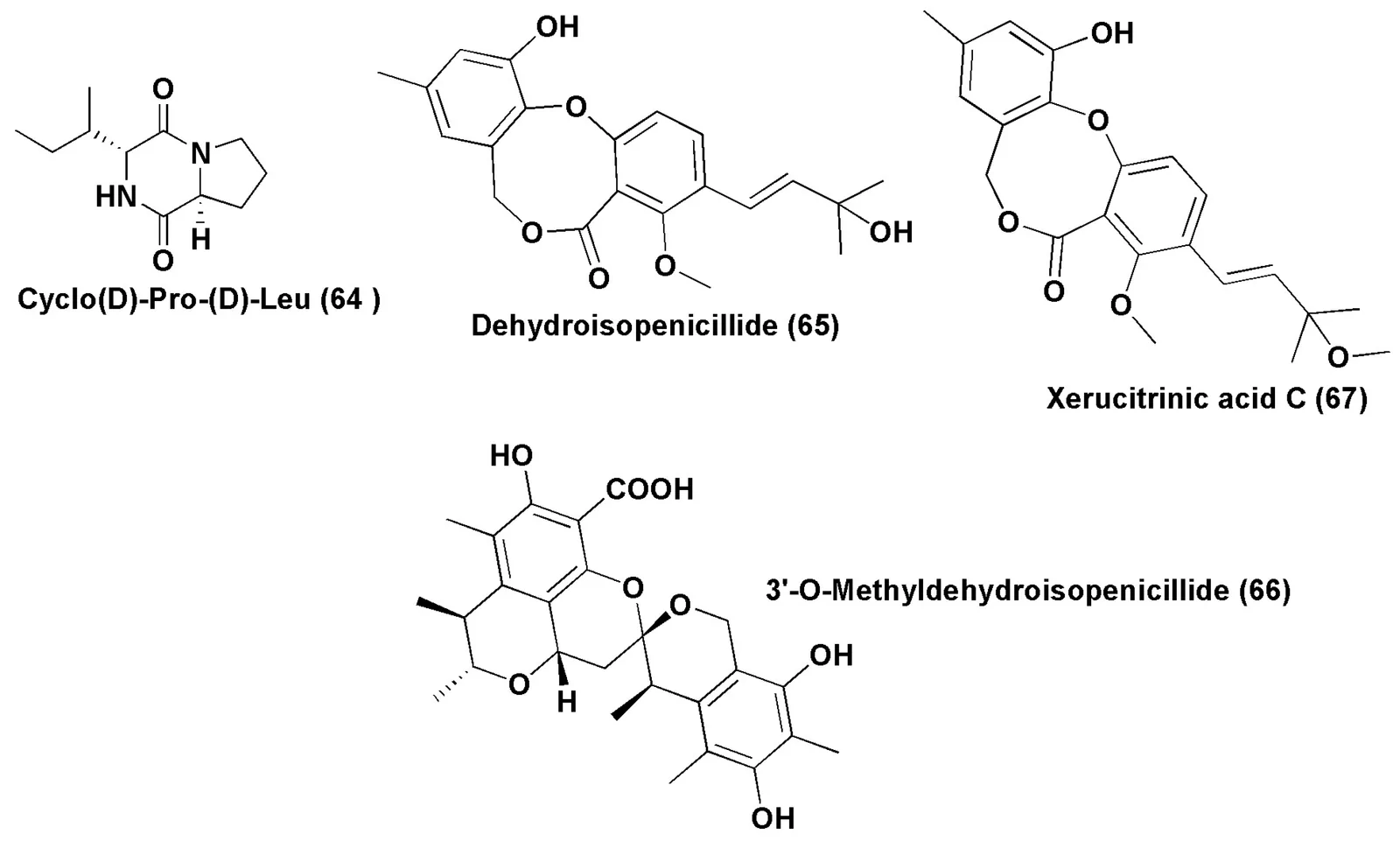
Anti-ESKAPE Metabolites from Mangrove-Associated *Talaromyces* spp.

Compounds penicillide (**62**) (Figure 3), cyclo(D)-Pro-(D)-Leu (**64**), dehydroisopenicillide (**65**), and 3′-O-methyldehydroisopenicillide (**66**) (Figure 4) were obtained from *Talaromyces* sp. SCSIO 41431, a fungus isolated from mangrove sediment collected at Gaoqiao Mangrove, Zhanjiang. These metabolites displayed antibacterial activity against *S. aureus*, with MIC values ranging between 25 and 50 μg/mL [76].

A novel dimeric citrinin derivative possessing an unusual spiro[chroman-2,3′-isochroman] framework, xerucitrinic acid C (**67**) (Figure 4), was characterized from the mangrove sediment-derived fungus *Talaromyces* sp. SCSIO 41428. This compound showed notable antibacterial effects against both *S. aureus* with MIC value of 25 μg/mL [77].

### 3.4. Anti-ESKAPE Metabolites from Diaporthe/Phomopsis spp.

**Figure 5:**
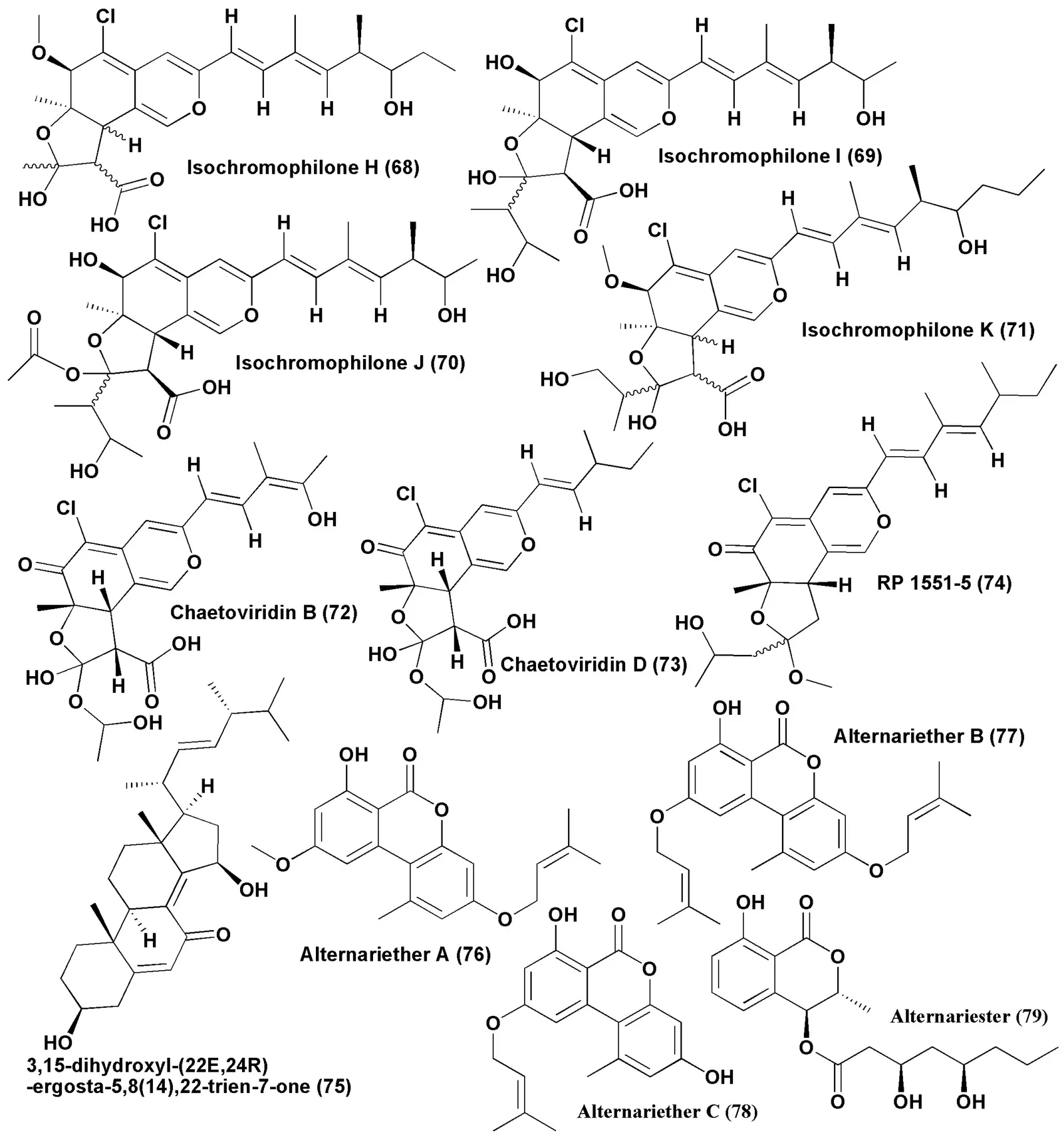
Anti-ESKAPE Metabolites from Mangrove-Associated *Phomopsis* spp., and *Alternaria* spp.

Several new metabolites, namely isochromophilones H–K (**68–71**), together with the previously reported compounds chaetoviridin B (**72**), chaetoviridin D (**73**), and RP 1551–5 (**74**) (Figure 5), were isolated from the endophytic fungus *Diaporthe perseae* associated with the mangrove plant *Pongamia pinnata* (L.) Pierre collected in Hainan, China. Among these metabolites, compounds **68–70** exhibited potent antibacterial activity against *S. aureus* (ATCC 35844), *Klebsiella pneumoniae* (ATCC BAA-1705), and *Pseudomonas aeruginosa* (ATCC BAA-2110), whereas compounds **71–74** showed comparatively weaker activity relative to the standard antibiotic ampicillin [78].

The known sterone derivative 3,15-dihydroxyl-(22E,24R)-ergosta-5,8(14),22-trien-7-one (**75**) (Figure 5) was isolated from the mangrove-associated fungus *Phomopsis* sp. MGF222. Compound **75** demonstrated moderate antibacterial activity against *S. aureus*, exhibiting an MIC value of 14.6 μM [79].

### 3.5. Anti-ESKAPE Metabolites from Alternaria spp.

Four new isocoumarin derivatives, alternariethers A–B (**76–77**) (Figure 5) alternariether C (**78**) and alternariester (**79**) (Figure 5), were isolated from the fermentation broth of *Alternaria malorum* FL39, a fungus obtained from *Myoporum bontioides* collected in the Leizhou Peninsula. These compounds exhibited weak to moderate antibacterial effects against *S. aureus* ATCC 6538, with MIC values ranging from 50 to 200 μg/mL. In comparison, the positive control cefradine showed stronger antibacterial activity with an MIC value of 3.125 μg/mL [80].

### 3.6. Anti-ESKAPE Metabolites from Other Fungi

**Figure 6:**
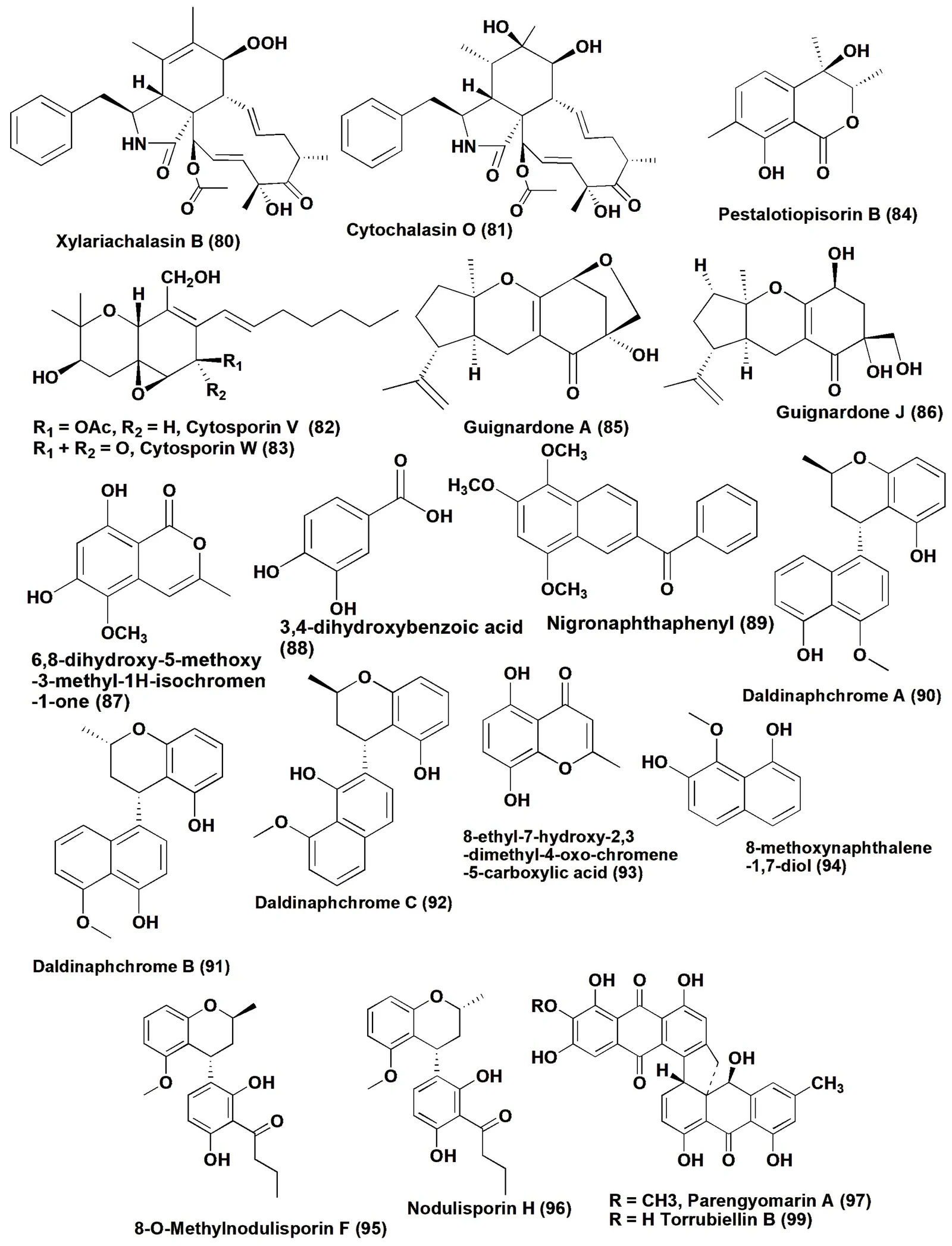

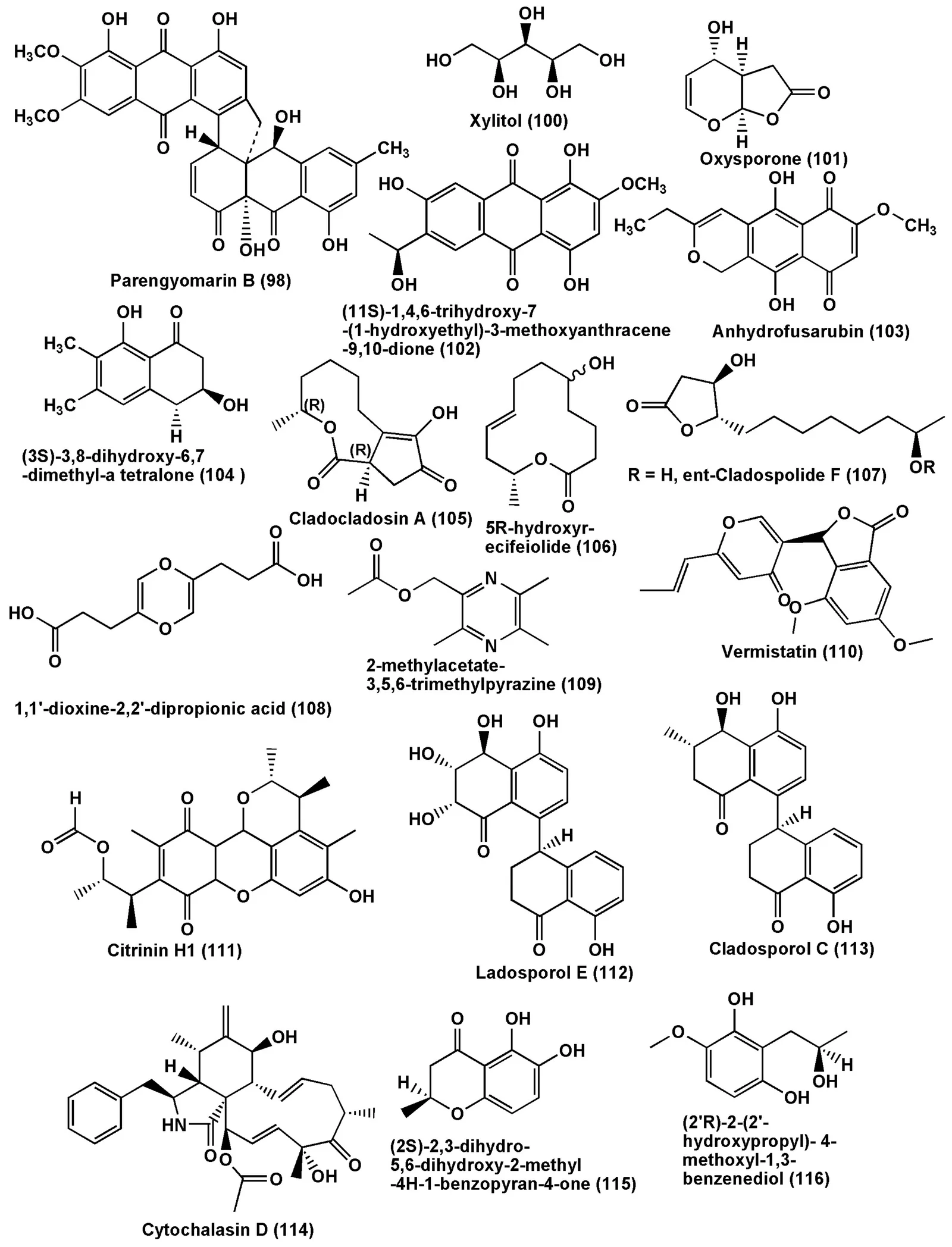

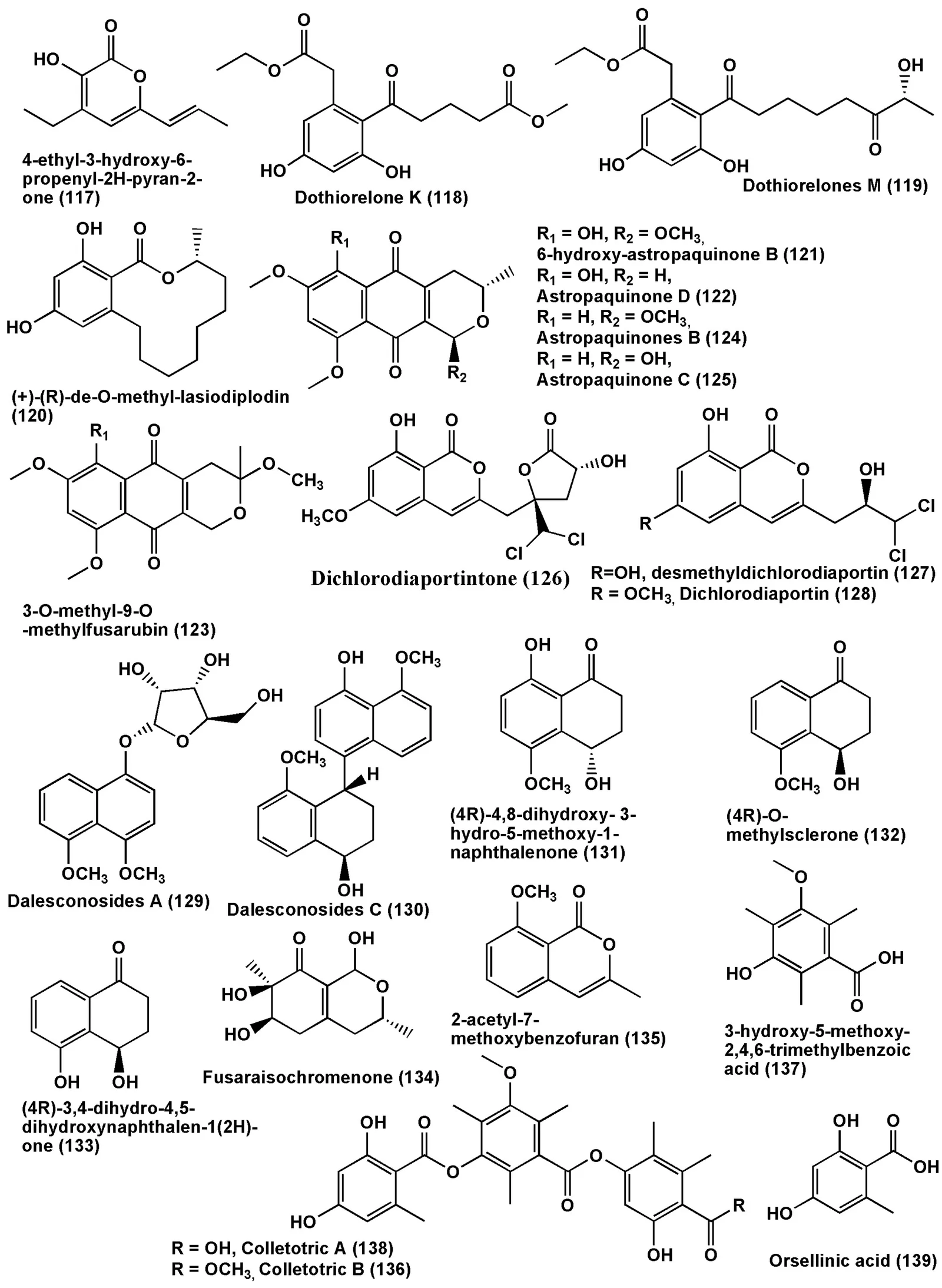
Anti-ESKAPE Metabolites from Mangrove-Associated other groups of Fungi.

Undescribed cytochalasin-type metabolites, xylariachalasin B (**80**), together with the known cytochalasin O (**81**) (Figure 6), were obtained from the mangrove-derived endophytic fungus *Xylaria arbuscula* QYF. This strain was isolated from the leaves of the mangrove plant *Kandelia candel*, collected from the Hainan Dongzhai Harbor Mangrove Reserve, China. Compound (**80**), characterized as a rare cytochalasin hydroperoxide, exhibited moderate antibacterial activity against *Staphylococcus aureus*, with an MIC of 12.5 μM. In contrast, compound (**81**) showed moderate inhibitory effects against *Pseudomonas aeruginosa*, also with an MIC value of 12.5 μM [81].

Cytosporins V (**82**) and W (**83**) (Figure 6) were isolated from *Pseudopestalotiopsis theae*, which was obtained from the roots of the mangrove plant *Rhizophora racemosa* collected near Lagos. Both compounds demonstrated antibacterial effects against drug-resistant *Acinetobacter baumannii* (ATCC BAA-1605) when tested in combination with a sublethal concentration of colistin (0.1 µM), with MIC values of 50 µM for (**82**) and 100 µM for (**83**), respectively [82].

A newly identified isocoumarin derivative, pestalotiopisorin B (**84**) (Figure 6), was reported from *Pestalotiopsis* sp., an endophytic fungus associated with the Chinese mangrove *Rhizophora stylosa* collected from Dong Zhai Gang Mangrove Garden, Hainan Island, China. This compound displayed moderate antibacterial activity against *P. aeruginosa*, with an MIC of 50 μg/mL, whereas ciprofloxacin, used as the positive control, showed a much stronger effect with an MIC of 0.078 μg/mL [83].

Chemical investigation of the endophytic fungus *Phyllosticta capitalensis*, isolated from *Bruguiera sexangula* collected in Dong Zhai Gang Mangrove Garden (Hainan Island, China), led to the identification of four meroterpenes: guignardone A (**85**), guignardone J (**86**), 6,8-dihydroxy-5-methoxy-3-methyl-1H-isochromen-1-one (**87**), and 3,4-dihydroxybenzoic acid (**88**) (Figure 6). Among these, compound (**85**) showed the strongest activity against *P. aeruginosa* with an MIC of 25 μg/mL. Compounds (**86**) and (**87**) exhibited weak inhibition against both *P. aeruginosa* and *S. aureus*, each with MIC values of 50 μg/mL, while compound (**88**) showed comparable activity against both organisms with MIC values of 25 μg/mL [84].

A novel compound, nigronaphthaphenyl (**89**) (Figure 6), was isolated from the mangrove-associated fungus *Nigrospora sphaerica*, obtained from *Bruguiera gymnorrhiza* collected at Attaragoda Wetland, Galle, Sri Lanka. This metabolite exhibited strong and selective antibacterial activity, particularly against Gram-positive *S. aureus* (ATCC 43300) and MRSA (ATCC 33591), with MIC values of 4 and 2 μg/mL, respectively, and also showed potent activity against Gram-negative *P. aeruginosa* (ATCC 27853) with an MIC of 2 μg/mL [85].

Three new naphthalene–chroman dimer derivatives, daldinaphchromes A–C (**90–92**), along with known metabolites 8-ethyl-7-hydroxy-2,3-dimethyl-4-oxo-chromene-5-carboxylic acid (**93**) and 8-methoxynaphthalene-1,7-diol (**94**) (Figure 6), were isolated from the mangrove-derived fungus *Daldinia eschscholzii* HJX1P2 collected in the Hainan Mangrove Conservation Area, Haikou. Compounds (**90–92**) showed antibacterial activity against *S. aureus* (ATCC 29213) with MIC values of 74.4, 74.4, and 148.8 μM, respectively. Compounds (**93**) and (**94**) were active against MRSA (ATCC 700699), with MIC values of 132.2 and 131.6 μM, respectively, and also inhibited MRSA (ATCC 43300) with MIC values of 390.6 and 263.2 μM, respectively. Vancomycin, used as the positive control, exhibited MIC values of 1.0 μM against *S. aureus* and both MRSA strains [86].

Two new polyketides, 8-O-methylnodulisporin F (**95**) and nodulisporin H (**96**) (Figure 6), were isolated from *Daldinia eschscholtzii* HJ004, which originated from the stem of the mangrove *Bruguiera sexangula* var. *rhynchopetala* collected in the South China Sea. Both compounds exhibited moderate antibacterial activity against *Staphylococcus aureus* and MRSA, with MIC values ranging from 6.25 to 12.5 μg/mL. Ciprofloxacin, used as a reference drug, showed stronger activity against *S. aureus* and MRSA with MIC values of 0.31 μg/mL, respectively [87].

Two torrubiellin-related derivatives, parengyomarin A (**97**) and parengyomarin B (**98**), along with the known compound torrubiellin B (**99**) (Figure 6), were obtained from the mangrove-associated fungus *Parengyodontium album*, isolated from *Avicennia marina* in Dong Zhai Gang Mangrove Garden, Hainan, China. Compounds (**97–99**) showed strong antibacterial activity against *S. aureus* and MRSA, with MIC values ranging from 0.39 to 3.12 μM. Moxifloxacin, used as a positive control, exhibited MIC values of 0.78 and 6.25 μM against *S. aureus* and MRSA, respectively [88].

xylitol (**100**) and oxysporone (**101**) (Figure 6) were isolated from *Pestalotia* sp. associated with the mangrove plant *Heritiera fomes* collected from the Sundarbans. Compound (**100**) showed antibacterial activity against multiple MRSA strains (XU212, ATCC25923, SA1199B, EMRSA-15, and 340702) with MIC values of 128, 128, 64, 64, and 128 μM, respectively. Compound (**101**) demonstrated slightly stronger activity, with MIC values of 128, 64, 32, 32, and 64 μM against the same strains. Norfloxacin, used as the positive control, exhibited MIC values of 16, 2, 32, 1, and 64 μM, respectively [89].

A new anthraquinone derivative, (11S)-1,4,6-trihydroxy-7-(1-hydroxyethyl)-3-methoxyanthracene-9,10-dione (**102**), together with the known compound anhydrofusarubin (**103**) (Figure 6), was obtained from the mangrove rhizosphere soil-derived fungus *Fusarium* sp. J3-2, which was isolated from soil collected in the Dongzhaigang Mangrove Natural Reserve, Hainan Province, China. Compound (**102**) showed weak to moderate antibacterial activity against five pathogenic bacterial strains, including *Staphylococcus aureus* ATCC 43300, ATCC 25923, ATCC 29213, and *E. faecium* ATCC 35667, with MIC values ranging from 25 to 50 μg/mL [90].

A known compound, (3S)-3,8-dihydroxy-6,7-dimethyl-α-tetralone (**104**) (Figure 6), was isolated from *Cladosporium* sp. JJM22 associated with *Ceriops tagal* collected from the South China Sea. This compound showed antibacterial activity against *S. aureus* and MRSA at a concentration of 20 μM [91].

A previously undescribed macrolide featuring an unusual bicyclo [5/9] ring system, named cladocladosin A (**105**) (Figure 6), was characterized from the mangrove-derived endophytic fungus *Cladosporium cladosporioides* MA-299, isolated from *Bruguiera gymnorrhiza* collected on Hainan Island. Compound (**105**) exhibited antibacterial activity against *Pseudomonas aeruginosa* with an MIC value of 4.0 μg/mL, compared with chloramphenicol, which showed an MIC of 2.0 μg/mL against the same strain [92].

Two polyketides, 5R-hydroxyrecifeiolide (**106**) and ent-cladospolide F (**107**) (Figure 6), were also isolated from *Cladosporium cladosporioides* MA-299 associated with *Bruguiera gymnorrhiza* collected from Hainan Island, China. Compound (**107**) displayed moderate antibacterial activity against *S. aureus* with an MIC of 8.0 μg/mL. In comparison, compound (**106**) showed activity against *P. aeruginosa* with an MIC value of 32 μg/mL [93].

A new pentenoic acid derivative, 1,1′-dioxine-2,2′-dipropionic acid (**108**), along with a new natural product, 2-methylacetate-3,5,6-trimethylpyrazine (**109**), and several known metabolites including vermistatin (**110**), citrinin H1 (**111**), cladosporol E (**112**), cladosporol C (**113**) (Figure 6), and cytochalasin D (**114**) (Figure 6), were obtained from *Cladosporium* sp. JS1-2, an endophytic fungus associated with *Ceriops tagal* collected from the South China Sea. These compounds exhibited antibacterial activity against *S. aureus* with MIC values of 25.0, 12.5, 25, 6.25, 1.56, 6.25, and 25 μg/mL, respectively, while ciprofloxacin served as the positive control with an MIC of 0.39 μg/mL [94].

Three new polyketides, (2S)-2,3-dihydro-5,6-dihydroxy-2-methyl-4H-1-benzopyran-4-one (**115**), (2′R)-2-(2′-hydroxypropyl)-4-methoxyl-1,3-benzenediol (**116**), and 4-ethyl-3-hydroxy-6-propenyl-2H-pyran-2-one (**117**) (Figure 6), were isolated from *Colletotrichum gloeosporioides* associated with *Ceriops tagal* collected from Dong Zhai Port, Hainan Province, China. These compounds showed antibacterial activity against *S. aureus* with MIC values of 12.5 μg/mL, while ciprofloxacin showed stronger activity with an MIC of 1.50 μg/mL [95].

Two cytosporone derivatives, dothiorelones K and M (**118–119**) (Figure 6), were isolated from the mangrove-derived fungus *Dothiorella* sp. ML002 associated with *Xylocarpus granatum* from the South China Sea. Both compounds exhibited antibacterial activity against *Staphylococcus aureus* ATCC 6538 with MIC values of 50 μg/mL [96].

(+)-(R)-de-O-methyl-lasiodiplodin (**120**) (Figure 6) was isolated from *Lasiodiplodia theobromae* M4.2-2 obtained from mangrove sediment collected at Dongzhai Harbor, Hainan, China. This compound showed antibacterial activity against *S. aureus* ATCC 29213 and ATCC 700699 with MIC values of 25 μg/mL [97].

Two new naphthoquinone derivatives, 6-hydroxy-astropaquinone B (**121**) and astropaquinone D (**122**), along with known compounds 3-O-methyl-9-O-methylfusarubin (**123**), astropaquinones B (**124**), and C (**125**) (Figure 6), were isolated from *Fusarium napiforme*, an endophytic fungus associated with *Rhizophora mucronata* collected in Takalar Regency, South Sulawesi Province, Indonesia. Compounds (**121–123**) showed moderate antibacterial activity against *S. aureus* NBRC 13276 with MIC values of 6.3, 12.5, and 6.3 μg/mL, respectively, and against *P. aeruginosa* ATCC 15442 with MIC values of 6.3 μg/mL. Compounds (124) and (**125**) exhibited activity against *S. aureus* YMF 3.17 with MIC values of 20 and 10 μg/mL, respectively [98].

A new isocoumarin derivative, dichlorodiaportintone (**126**), along with known analogues desmethyldichlorodiaportin (**127**) and dichlorodiaportin (**128**) (Figure 6), were isolated from *Ascomycota* sp. CYSK-4 associated with *Pluchea indica* collected from Shankou Mangrove Nature Reserve, Guangxi Province, China. Compounds (**127–128**) showed antibacterial activity against *S. aureus* and *Klebsiella pneumoniae* with MIC values ranging between 25 and 50 μg/mL. Compound (**126**) also exhibited activity against both strains with MIC values of 50 μg/mL [99].

New naphthalene derivatives dalesconosides A (**129**) and C (**130**), along with known compounds including (4R)-4,8-dihydroxy-3-hydroxy-5-methoxy-1-naphthalenone (**131**), (4R)-O-methylsclerone (**132**), (4R)-3,4-dihydro-4,5-dihydroxynaphthalen-1(2H)-one (**133**), fusaraisochromenone (**134**), and 2-acetyl-7-methoxybenzofuran (**135**), (3S)-3,8-dihydroxy-6,7-dimethyl-α-tetralone (**104**), (Figure 6), were isolated from *Daldinia eschscholzii* MCZ-18 associated with *Ceriops tagal* collected from Dong Zhai Gang Mangrove Garden, Hainan Island, China. These compounds showed varying antimicrobial activities against *P. aeruginosa*, and MRSA, with MIC values ranging from 6.25 to 25 μg/mL, while ciprofloxacin exhibited significantly stronger activity [100]. Five polyketides, including colletotric B (**136**), 3-hydroxy-5-methoxy-2,4,6-trimethylbenzoic acid (**137**), along with known analogues colletotric A (**138**) and orsellinic acid (**139**) (Figure 6), were isolated from *Phoma* sp. SYSU-SK-7 associated with *Kandelia candel* collected from Shankou Mangrove Nature Reserve, Guangxi Province, China. Compound (**138**) showed strong activity against MRSA with an MIC of 6.28 μg/mL. Compound (**136**) exhibited potent antibacterial activity against *P. aeruginosa* and MRSA with MIC values of 1.67 and 3.36 μg/mL, respectively, while compound (**139**) was active against *P. aeruginosa* with an MIC of 2.10 μg/mL [101].

## 4. Future Directions

Achieving good health and well-being remains one of the fundamental objectives of the United Nations Sustainable Development Goals (SDGs), with the global community striving toward this target by 2030 [102]. Nevertheless, the burden of infectious and non-infectious diseases continues to threaten public health worldwide. Despite significant advances in medicine, many diseases still lack effective therapeutic interventions, and even when treatment options exist, prolonged use of conventional drugs may result in adverse effects, reduced efficacy, or the emergence of drug resistance [103,104]. The recent rise in antimicrobial resistance (AMR), particularly among multidrug-resistant ESKAPE pathogens, has further exposed the limitations of the current antimicrobial arsenal. In this context, there is an urgent need to identify innovative, sustainable, and biologically relevant sources of novel therapeutic agents.

Natural products have historically played a pivotal role in drug discovery, providing the foundation for many clinically important antibiotics, anticancer agents, and immunosuppressive compounds [105]. However, although terrestrial plants have long been recognized as reservoirs of medicinal compounds, their associated microbial communities remain comparatively underexplored. Increasing evidence suggests that microorganisms inhabiting unique ecological niches possess remarkable biosynthetic capabilities, enabling them to produce structurally diverse metabolites with significant pharmacological potential [106,107]. Among these microbial resources, mangrove-associated endophytic fungi represent an especially promising yet underutilized source of bioactive compounds.

The ecological complexity of mangrove ecosystems creates highly selective environmental conditions characterized by fluctuations in salinity, oxygen availability, temperature, and nutrient status. Such stressful habitats are likely to drive microbial adaptation and metabolic innovation, resulting in the production of unique secondary metabolites not commonly encountered in organisms from less extreme environments. It is therefore reasonable to speculate that mangrove-associated fungi have evolved sophisticated chemical defense systems that could be exploited in the search for novel anti-infective agents. However, despite increasing reports describing the isolation of biologically active metabolites from these fungi, our understanding of their ecological roles and biosynthetic significance remains incomplete.

It has been estimated that hundreds of thousands of plant species exist worldwide, each harboring diverse endophytic microbial communities that remain largely uncharacterized [108]. The diversity of these microbial populations offers a vast and largely untapped repository of genetic and biochemical resources. Different endophytic fungi exhibit varying host specificities and colonization strategies, suggesting that particular host–microbe associations may influence the nature and diversity of the metabolites produced [109]. From a drug discovery perspective, this presents an extraordinary opportunity to identify niche-specific metabolites with enhanced biological activity against priority pathogens, including members of the ESKAPE group.

Although the pharmaceutical relevance of fungal endophytes has received growing attention, research efforts have predominantly focused on the initial discovery of bioactive compounds rather than their progression along the drug development pipeline [110,111,112]. Numerous studies have demonstrated that endophytic fungi can synthesize metabolites possessing antimicrobial, anticancer, antioxidant, antiviral, and anti-inflammatory properties [113,114]. Yet only a limited number of these compounds have advanced to detailed pharmacological characterization or preclinical evaluation. In our opinion, future investigations should prioritize translational approaches aimed at transforming promising fungal metabolites into viable therapeutic candidates [115,116].

Particular emphasis should be placed on compounds exhibiting activity against ESKAPE pathogens, which continue to pose serious threats in healthcare settings due to their exceptional capacity to evade existing antibiotics. Rather than relying solely on traditional antimicrobial screening methods, future research should incorporate assays designed to evaluate anti-biofilm activity, quorum sensing inhibition, resistance-modifying effects, and synergistic interactions with established antibiotics. Such strategies may uncover metabolites capable of restoring the effectiveness of current antimicrobial agents or targeting virulence mechanisms that contribute to persistent infections.

The discovery of novel antibacterial agents from mangrove-associated fungi is increasingly benefiting from the integration of advanced genomic, metabolomic, computational, and biotechnological approaches. Traditional bioactivity-guided isolation methods often encounter limitations due to the repeated identification of known metabolites and the inability to access cryptic biosynthetic pathways under standard laboratory conditions. Therefore, modern strategies aimed at activating, predicting, and optimizing fungal secondary metabolism are becoming essential for expanding the chemical diversity of antimicrobial candidates. Genome mining, metagenomics, transcriptomics, and metabolomics can facilitate the identification of cryptic biosynthetic gene clusters that remain silent under conventional laboratory conditions. Likewise, approaches such as co-cultivation, epigenetic modification, and the “one strain-many compounds” (OSMAC) strategy have demonstrated considerable promise in stimulating the production of previously inaccessible metabolites.

Adaptive laboratory evolution (ALE) is an another widely used strategy in synthetic biology for generating novel biological and phenotypic traits and driving strain improvement [117,118]. In filamentous fungi, however, ALE remains comparatively underdeveloped relative to bacteria and yeasts, largely because of their complex multicellular growth patterns, long generation times, genomic plasticity, and the difficulty of sustaining stable evolutionary trajectories over successive passages. In the context of fungal natural product discovery, ALE offers a valuable complement to established strategies such as OSMAC (One Strain–Many Compounds), epigenetic modulation, genome mining, and metabolic engineering, as it can drive the evolution of strains with enhanced expression of otherwise silent biosynthetic gene clusters (BGCs). Because fungal genomes typically harbor numerous cryptic secondary metabolite clusters that remain transcriptionally inactive under standard laboratory conditions, selective pressure applied during ALE can favor mutations in global regulatory networks including the LaeA/VeA/Velvet complex as well as in transcription factors, chromatin remodelling machinery, transporter systems, and precursor-supply pathways. Such mutations can unlock chemical diversity that would otherwise remain inaccessible [119]. The development of bioinformatic platforms such as antiSMASH has significantly improved BGC prediction by enabling rapid identification, annotation, and comparison of secondary metabolite gene clusters across microbial genomes. Integration of antiSMASH analysis with metabolomics platforms, particularly high-resolution LC–MS/MS, molecular networking, and Global Natural Products Social Molecular Networking (GNPS), has facilitated the correlation of predicted biosynthetic pathways with experimentally detected metabolites, thereby accelerating the discovery of novel antibacterial compounds [120,121]. When integrated with whole-genome sequencing (WGS), transcriptomics, metabolomics, and CRISPR-based genome engineering, ALE becomes a powerful platform for pinpointing the adaptive mutations underlying improved biosynthetic phenotypes, ultimately guiding rational and targeted strain engineering efforts. Integrating these technologies into mangrove fungal research could significantly expand the repertoire of bioactive molecules available for antimicrobial development.

The integration of artificial intelligence (AI), machine learning (ML), and computational chemistry provides new opportunities to accelerate antibacterial drug discovery. AI-based algorithms can analyse large chemical and biological datasets to predict antimicrobial activity, toxicity, pharmacokinetic properties, and structure–activity relationships of fungal metabolites. Machine learning models trained in natural product databases can prioritize promising compounds before experimental validation, reducing time and resource requirements. Similarly, molecular docking and molecular dynamics simulations allow prediction of interactions between fungal-derived compounds and bacterial targets, including enzymes involved in cell wall synthesis, DNA replication, and antibiotic resistance pathways. These computational approaches are increasingly being combined with experimental screening to improve the identification of lead molecules [122,123].

Another important consideration involves the sustainable exploitation of mangrove ecosystems. As interest in mangrove-derived metabolites increases, there is a corresponding responsibility to ensure that bioprospecting activities do not compromise the ecological integrity of these vulnerable habitats. Conservation-oriented sampling practices, coupled with ex situ cultivation techniques and microbial preservation programs, will be essential for balancing scientific advancement with environmental stewardship. The protection of mangrove biodiversity should therefore be regarded not only as an ecological priority but also as an investment in future pharmaceutical innovation [124].

Furthermore, the production of microbial metabolites offers several advantages over direct extraction from medicinal plants. Microbial cultivation can potentially provide more consistent yields, reduced harvesting pressure on plant resources, and opportunities for fermentation-based optimization. By manipulating endophyte communities and understanding their interactions with host plants, it may be possible to enhance metabolite production or induce the synthesis of entirely new compounds [113]. Synthetic biology and metabolic engineering approaches could further facilitate scalable production processes, thereby improving the commercial feasibility of fungal-derived therapeutics.

Beyond discovery, nanotechnology-based delivery systems and combination therapies offer promising approaches for improving the clinical translation of fungal-derived antibacterial compounds. Nanoparticle-based formulations can enhance compound solubility, stability, bioavailability, and targeted delivery while reducing toxicity. Furthermore, combining fungal metabolites with conventional antibiotics may restore antibiotic efficacy by inhibiting resistance mechanisms such as efflux pumps, biofilm formation, or enzymatic antibiotic degradation. Such synergistic approaches could provide effective strategies against multidrug-resistant ESKAPE pathogens.

Despite these encouraging prospects, significant challenges remain. Methodological limitations, difficulties in fungal cultivation, low metabolite yields, complex purification procedures, and economic considerations continue to hinder the advancement of fungal natural products into clinical development. Additionally, the preference for synthetic drugs within pharmaceutical industries often reflects concerns related to reproducibility, regulatory approval, and large-scale manufacturing. Addressing these barriers will require interdisciplinary collaboration among microbiologists, natural product chemists, pharmacologists, bioinformaticians, clinicians, and industrial stakeholders.

In conclusion, mangrove-associated endophytic fungi represent an exceptional but insufficiently explored resource for the discovery of novel bioactive compounds. Their unique ecological adaptations and biosynthetic versatility position them as promising contributors to the global search for new antimicrobial agents, particularly against multidrug-resistant ESKAPE pathogens. Future research should move beyond descriptive studies toward mechanism-based investigations, translational validation, and sustainable exploitation strategies. By combining advances in omics technologies, innovative cultivation methods, and collaborative research frameworks, the therapeutic potential of mangrove fungal metabolites may be fully realized. In our view, harnessing these microbial resources is not merely an academic endeavor but a public health imperative that could contribute meaningfully to addressing the escalating crisis of antimicrobial resistance.

## 5. Conclusions

Mangrove-associated fungal endophytes represent a promising and largely untapped source of structurally diverse bioactive metabolites with significant therapeutic potential against ESKAPE pathogens. The increasing prevalence of antimicrobial resistance among these clinically important microorganisms has intensified the search for alternative strategies to supplement or replace conventional antibiotics. In this regard, endophytic fungi inhabiting mangrove ecosystems offer a unique opportunity for the discovery of novel compounds with distinctive chemical scaffolds and mechanisms of action that may overcome existing resistance barriers. Although mangrove-associated fungi have demonstrated promising activity against ESKAPE pathogens, current evidence is disproportionately focused on Gram-positive pathogens, particularly S. aureus and MRSA. Comprehensive evaluation against Gram-negative ESKAPE members remains an important future research priority.

The findings discussed in this review highlight the remarkable capacity of mangrove-derived fungal endophytes to produce metabolites exhibiting antibacterial activities relevant to the management of infections caused by multidrug-resistant pathogens. Although numerous mangrove fungal metabolites have demonstrated antibacterial activity, their translation into therapeutic agents remains challenging. A considerable proportion of reported compounds exhibit MIC values in the range of 25–100 μg/mL, indicating moderate activity that requires further optimization. In contrast, compounds displaying low micromolar MIC values, broad-spectrum activity against resistant pathogens, favourable selectivity indices, and acceptable cytotoxicity profiles represent more realistic lead candidates. Beyond antibacterial potency, successful drug development requires evaluation of pharmacokinetic properties, metabolic stability, toxicity, production yield, and scalability. Many fungal metabolites are isolated in limited quantities, creating challenges for preclinical development; therefore, approaches such as pathway engineering, heterologous expression of biosynthetic gene clusters, fermentation optimization, and chemical derivatization are essential for improving accessibility and therapeutic potential.

Furthermore, this review emphasizes that natural product-based drug discovery has evolved into a highly interdisciplinary endeavor that extends beyond the traditional domains of microbiology and natural product chemistry. The successful development of endophyte-derived therapeutics requires the collective expertise of taxonomists, molecular biologists, chemical ecologists, biochemists, agronomists, bioengineers, pharmacologists, and medicinal chemists. Through collaborative approaches and the integration of advanced technologies such as genomics, metabolomics, synthetic biology, and computational modeling, the pace of antimicrobial discovery can be significantly accelerated.

Importantly, the exploration of mangrove-associated endophytes aligns with the broader objectives of promoting good health and well-being as outlined in the United Nations Sustainable Development Goals. However, translating promising fungal metabolites into clinically relevant drugs remains a long-term undertaking that demands sustained investment, strategic partnerships, and a commitment to the conservation of mangrove ecosystems. Nevertheless, the growing body of evidence supporting the therapeutic potential of these microorganisms suggests that they may constitute an invaluable resource in the global fight against antimicrobial resistance. Continued exploration of these hidden microbial reservoirs may ultimately lead to the development of innovative therapeutics capable of addressing one of the most pressing public health challenges of the twenty-first century.

## Data Availability

No new data were created or analyzed in this study. Data sharing is not applicable to this article.

## Supplementary Materials

The following supporting information can be downloaded at: https://www.mdpi.com/article/10.3390/life16081272/s1, Table S1: Anti ESKAPE compounds isolated from Mangrove-Associated Fungi.
