# Supplementary material for: Bioactive Compounds from Mangrove-Associated Fungi as Leads Against ESKAPE Pathogens
Source: Life (Basel). 2026 Jul 31;16(8):1272. doi: 10.3390/life16081272 (PMC13514675; doi:10.3390/life16081272)
Supplement: Supplementary file 1 [file life-16-01272-s001.zip › life-4404526-supplementary.pdf]

**Supplementary Table S1** Anti ESKAPE compounds isolated from Mangrove-Associated Fungi

| Sr. No. | Compound Name | Chemical class | Fungi | Host plant | Target ESKAPE pathogen | MIC/$IC_{50}$ values | Refs. |
|---|---|---|---|---|---|---|---|
| 1. | 2-Deoxy-sohirnone C (1) | Sorbicillin derivative | *Penicillium* sp. GD6 | *Bruguiera gymnorrhiza* | MRSA | MIC - 80 µg/mL | [49]. |
| 2. | Penibenzophenone A (2) | Chlorinated benzophenone | *Penicillium citrinum* HL-5126 | *Bruguiera sexangula* var. *rhynchopetala* | *S. aureus* | MIC - 20 µg/mL | [50]. |
| 3. | Penicilactone A (3) | Lactones | *Penicillium* sp. TGM112 | *B. sexangula* var. *rhynchopetala* South China Sea | *S. aureus* | MIC - 6.25 µg/mL | [51]. |
| 4. | Penicilones G and H (4–5), | Brominated azaphilones | *Penicillium janthinellum* HK1-6, | Rhizosphere soil collected in the Dongzhaigang Mangrove Natural Reserve, Hainan Island | *S. aureus* strains (ATCC 43300, 33591, 29213, and 25923), *Enterococcus faecalis* ATCC 51299, and *E. faecium* ATCC 35667, | MIC values range from 3.13 to 25 µg/mL. | [52]. |
| | Penijanthinones A (6), | Tricyclic polyketides | | | *S. aureus* (ATCC 43300), *S. aureus* (ATCC 33591), *S. aureus* (ATCC 29213), *E. faecalis* (ATCC 51299), and *E. faecium* (ATCC 35667) | MIC values range from 3.13 to 25 µg/mL. | |
| | (S,E)-2,4-dimethyldec-2-enoic | Ester | | | MRSA ATCC | MIC - 3.13 | |

| | | | | | | | |
|---|---|---|---|---|---|---|---|
| | acid (**7**) | | | | 33591<br>*S. aureus* ATCC 43300 and ATCC 33591, | μg/mL<br>MIC - 25 μg/mL each | |
| 5. | Sch725680 (8) | Azaphilone | *Penicillium pinophilum* SCAU037 | Sediment derived from the roots of *Rhizophora stylosa* collected on Techeng Isle, China | *S. aureus*, | $IC_{50}$ - 2.6 μM | [53]. |
| 6. | Peninaphones A–C (9–11) | Naphtho-γ-pyrones | *Penicillium* sp. HK1-22 | Mangrove rhizosphere soil from Dongzhaigang Mangrove Natural Reserve, Hainan Island. | *S. aureus* strains (ATCC 43300, 33591, 29213, and 25923) | MIC values range from 12.5 to 50 μg/mL. | [54]. |
| 7. | Peninaphone A (9) and peninaphone B (10) | Naphtho-γ-pyrones | *Penicillium* sp. HK1-22 | Mangrove rhizosphere soil from Dongzhaigang Mangrove Natural Reserve, Hainan Island. | *S. aureus* (ATCC 33591 and 43300), sensitive *S. aureus* (ATCC 29213 and 25923), *E. faecium* ATCC 35667, and *E. faecalis* ATCC 51299. | Inhibition zones ranging from 10.4 to 21.0 mm | [55]. |
| 8. | Emindole SB (12) | Indole diterpenes | *Penicillium javanicum* HK1-23 | Rhizosphere soil from Dongzhaigang Mangrove Natural Reserve, Hainan Island | *S. aureus* ATCC 33591 | MIC - 6.25 μg/mL. | [56]. |
| | Penialidin C (13) | Polyketides | | | *S. aureus* strains ATCC 43300, 33591, 25923, and 29213, with | MIC values range from 0.78 to 25 μg/mL. | |

| | | | | | | | |
|---|---|---|---|---|---|---|---|
| | Penialidin A (14) | Polyketides | | | *S. aureus* ATCC 43300 and ATCC 29213 | MIC - 3.13 and 12.5 µg/mL, | |
| 9 | Penibenzophenone C (15) and Penibenzophenone D (16) | Benzophenone | *Penicillium* species | *Acanthus ilicifolius* collected from the South China Sea | MRSA | MIC - 3.12 and 6.25 µg/mL | [57]. |
| 10. | Phomoindene A (17) | Indane class | *Penicillium verruculosum* TGM14 | *Xylocarpus granatum* collected from the South China Sea. | *S. aureus* | MIC - 50 µg/mL | [58]. |
| 11. | Peniazaphilone A (18) | Azaphilones derivatives | Co-cultivation *Penicillium sclerotiorum* THSH-4 and *Penicillium sclerotiorum* ZJHJJ-18 | *Aegiceras corniculatum* collected from the Huizhou East Mangrove National Nature Reserve in Guangdong Province, China | *Pseudomonas aeruginosa* and MRSA, | MIC - 12.5 µM/mL | [59]. |
| 12. | GKK1032C (19) and 4-hydroxy-17R-methylincisterol (20) | Alkaloid<br><br>Sterols | *Penicillium steckii* SCSIO 41025 | *Avicennia marina* collected from Zhanjiang, Guangdong Province, China. | *S. aureus* | MIC - 3.9 and 7.8 µg/mL, | [60]. |
| 13. | Penicimarin G (21) | Dihydroisocoumarin | *Penicillium verruculosum* | *Xylocarpus granatum* collected from the South China Sea | *S. aureus* | MIC - 25 µg/mL | [61]. |
| 14. | Peniprenylphenol A (22), | Tetrasubstituted benzene derivative | *Penicillium chrysogenum* ZZ1151 | Mangrove sediment from the tidal flats of | MRSA | MIC - of 6 µg/mL. | [62]. |

| | | | | | | | |
|---|---|---|---|---|---|---|---|
| | Penicimumide (23), | alkaloid | | Pangkep District, South Sulawesi Province, Indonesia | *E. coli* | 13 mg/mL | |
| | Preparaherquamide (24), uridine (25), 1,2-Seco-trypacidin (27), 4-Hydroxybenzeneacetic acid methyl ester (30), | Prenylated indole alkaloids (24), Pyrimidine nucleosides (25) Polyketide (27) Phenolic esters and phenylacetates (30) | | | MRSA | MIC values range from 7 to 25 mg/mL. | |
| | Thymine (26), Clavatol (29), | Pyrimidine nucleobase (26) Polyketide (29) | | | MRSA | MIC - 25 mg/mL | |
| | 2-Hydroxyphenylacetic acid methyl ester (31), | Phenol and aromatic ester | | | *MRSA* | MIC - 13 µg/mL | |
| | Communol G (28), 4-Hydroxyphenylethanone (32) | Labdane diterpenoid and aromatic polyketide (28), Alkyl-phenylketones (32) | | | MRSA | *MIC: 25 mg/mL* | |
| 15. | Annularins L and M (33–34) | α-Pyrone derivatives | *Penicillium herquei* MA-370, | Rhizospheric soil of *Rhizophora mucronata* from Hainan Island, China. | *Pseudomonas aeruginosa* QDIO-4 | MIC - 64 µg/mL. | [63]. |
| **Anti-ESKAPE Metabolites from *Aspergillus* spp** | | | | | | | |
| 16 | 5-Epi-asperdichrome (35) | Tetrahydroxanthone dimers | *Aspergillus versicolor* HDN1009 | Mangrove-associated soil collected in Guangzhou, China | *Pseudomonas aeruginosa* | MIC - 100 µM. | [64]. |

| | | | | | | | |
|---|---|---|---|---|---|---|---|
| 17. | Asperphenone A-B (36–37) | Phenone derivatives | *Aspergillus sp.* YHZ-1 | Mangrove-derived fungus | *Staphylococcus aureus* CMCC(B) 26003, *Streptococcus pyogenes* ATCC19615, *Bacillus subtilis* CICC 10283, and *Micrococcus luteus*, | MIC values range from32.0 to 64.0 µM | [65]. |
| 18. | (7S,10S)-7,10-Epoxysydonic acid (38), (7R,11S)-7,12-epoxysydonic acid (39), 7-Deoxy-7,14-didehydro-12-hydroxysydonic acid (40), and (E)-7-Deoxy-7,8-didehydro-12-hydroxysydonic acid (41), Engyodontiumone I (42), (+)-Hydroxysydonic acid (43), and (−)-(7S)-10-Hydroxysydonic acid (44) | Phenolic bisabolane sesquiterpenoids | *Aspergillus* sp. xy02 | *Xylocarpus moluccensis* was collected in Trang Province, Thailand. | *S. aureus* ATCC 25923, | IC50 - 32.2, 36.0, 41.9, 31.5, 33.4, 34.0, and 36.3 µM, | [66]. |
| 19. | 2-(hydroxymethyl)-3-propylphenol (45) and (−)-brassicadiol (46) | Phenylpropanoid analogue (45), benzofuran (46) | *Aspergillus sp.* ZJ-68 | *Kandelia candel* collected in the Zhanjiang Mangrove Nature Reserve, Guangdong Province, China | *S. aureus* | MIC values range from4.15 to 12.5 µg/mL | [67] |

| | | | | | | | |
|---|---|---|---|---|---|---|---|
| 20. | Sonneratinone (47) | Benzofuranone derivative | *Aspergillus niger* | *Sonneratia apetala* collected from the Sundarbans. | *S. aureus*<br>*P. aeruginosa* (NCTC 12903) | $2.5 \times 10^{-1}$ mg/mL<br>$5 \times 10^{-1}$ mg/mL | [68]. |
| 21. | Prenylterphenyllins I (48) and J (49) | p-Terphenyls, | *Aspergillus candidus* LDJ-5 | *Rhizophora apiculata* collected in Sanya Bailu Park, Hainan Province, China | *P. aeruginosa* | MIC - 42 and 90 µg/mL. | [68]. |
| | | | | | | MIC - 0.52 µg/mL | |
| 22. | Asperbutenolide A (50), | Aromatic butenolide | *Aspergillus terreus* SCAU011 | rhizosphere soil of the mangrove plant *Rhizophora stylosa*. | *S. aureus* and *Vibrio splendidus* | IC50 - 1.3 and 3.7 µM | [69]. |
| | Butyrolactone I (51) | Aromatic butenolide | | | *S. aureus* | IC50 - 13.5 µM. | |
| 23. | Eugenitol (52) | Polyketide derivative | *Aspergillus* sp. SCSIO41407 | sediment collected near the Hongsha River estuary in the South China Sea, Sanya City, Hainan Island. | MRSA | MIC - 485.4 µM | [70]. |
| 24. | Fumigaclavine C (53), Azaspirofuran B (54), Fraxetin (55) | Ergot alkaloid, Hetero-spirocyclic γ-lactam, O-methylated hydroxycoumarin | *Aspergillus fumigatus* | *Ceriops decandra* collected from the Sundarbans | *EToAc Extract*<br>*S. aureus* and *P. aeruginosa*, | MIC - 0.625 and 0.156 mg/mL, | [71]. |
| 25. | Aflaxanthones A (56) and B (57) | Heterodimeric tetrahydroxanthones | *Aspergillus flavus* QQYZ | *Kandelia candel* collected in Huizhou, Guangdong Province, China | MRSA | MIC - 12.5 µM | [72]. |

| | | | | | | | |
|---|---|---|---|---|---|---|---|
| 26. | Asperazine (58), aurasperone B (59), aurasperone F (60), TMC-256A1 (61), | Indole alkaloid, Dinaphthopyrone, Dinaphthopyrone, Polyketide, | *Aspergillus sp.* IQ-548 | mangrove soil, leaves, and roots of *Rhizophora mangle* and *Laguncularia racemosa* collected from lagoons in Oaxaca, Mexico. | *Acinetobacter baumannii* A564 | IC50 - 6.9, 9.9, 8.3, and 7.4 µg/mL | [73]. |
| 27. | Penicillide (62) | Diarylethers | Talaromyces sp. IQ-567 | mangrove soil, leaves, and roots of *Rhizophora mangle* and *Laguncularia racemosa* collected from lagoons in Oaxaca, Mexico. | multidrug-resistant *Acinetobacter baumannii* A564 | 40% inhibition at 100 µg/mL | [73]. |
| 28. | Ganodermasides D (63) | | *Aspergillus terreus* GXIMD 03158 | *Acanthus ilicifolius*. | *S. aureus*, | MIC - 6.25 µg/mL | [74] |
| **Anti-ESKAPE Metabolites from Talaromyces spp.** | | | | | | | |
| 29. | Cyclo(D)-Pro-(D)-Leu (64), Penicillide (62), Dehydroisopenicillide (65), and 3′-O-Methyldehydroisopenicillide (66) | Diketopiperazines, Polyketide, Polyketide, Polyketide | *Talaromyces* sp. SCSIO 41431 | mangrove sediment collected at Gaoqiao Mangrove, Zhanjiang. | *S. aureus* | MIC values range from 25 and 50 µg/mL | [75]. |
| 30 | Xerucitrinic acid C (67) | Dimeric citrinin derivative | *Talaromyces* sp. SCSIO 41428. | | *S. aureus* and *Streptococcus suis*, | MIC - 25 µg/mL | [76]. |
| **Anti-ESKAPE Metabolites from Diaporthe/Phomopsis spp.** | | | | | | | |
| 31. | Isochromophilones H–J (68-70), | Azaphilone derivative | *Diaporthe perseae* | *Pongamia pinnata* collected in Hainan, China | *S. aureus* (ATCC 35844), *Klebsiella pneumoniae* | Potent activity relative to the standard | [77]. |

| | | | | | | | |
|---|---|---|---|---|---|---|---|
| | | | | | (ATCC BAA-1705), and *Pseudomonas aeruginosa* (ATCC BAA-2110), | antibiotic ampicillin | |
| | Isochromophilones K (71), Chaetoviridin B (72), Chaetoviridin D (73), and RP 1551–5 (74) | Azaphilone derivative, Azaphilone derivative, Azaphilone derivative, Azaphilone derivative, | | | | Comparatively weaker activity relative to the standard antibiotic ampicillin | |
| 32. | 3,15-Dihydroxyl-(22E,24R)-ergosta-5,8(14),22-trien-7-one (75) | Sterone derivatives, | *Phomopsis* sp. MGF222 | mangrove-associated fungus | *S. aureus*, | MIC - 14.6 μM | [78]. |
| **Anti-ESKAPE Metabolites from Alternaria spp.** | | | | | | | |
| 33. | Alternariethers A–B *(76-77),* Alternariether C (78) and Alternariester (79), | Isocoumarins | *Alternaria malorum FL39* | Myoporum bontioides *collected in the Leizhou Peninsula* | S. aureus *ATCC 6538,* | MIC values range from 50 to 200 μg/mL | *[79]* |
| **Anti-ESKAPE Metabolites from other Fungi** | | | | | | | |
| 34, | Xylariachalasin B (80), | Cytochalasins | *Xylaria arbuscula* QYF. | *Kandelia candel*, collected from the Hainan Dongzhai Harbor Mangrove Reserve, China. | *Staphylococcus aureus* | MIC - 12.5 μM | [80]. |
| | Cytochalasin O (81) | Cytochalasins | | | *Pseudomonas aeruginosa* | MIC - 12.5 μM | |
| 35. | Cytosporins V (82) and W (83) | Polyketide derivatives | *Pseudopestalotiopsis theae* | *Rhizophora racemosa* collected near Lagos. | *Acinetobacter baumannii* (ATCC BAA-1605) | MIC - 50 μM and 100 μM | [81]. |

| | | | | | | | |
|---|---|---|---|---|---|---|---|
| 36. | Pestalotiopisorin B (84) | Isocoumarin derivative | *Pestalotiopsis* sp., | *Rhizophora stylosa* collected from Dong Zhai Gang Mangrove Garden, Hainan Island, China | *P. aeruginosa* | MIC - 50 µg/mL | [82]. |
| 37. | Guignardone A (85), | Meroterpenes | *Phyllosticta capitalensis* | *Bruguiera sexangula* collected in Dong Zhai Gang Mangrove Garden (Hainan Island, China) | *P. aeruginosa* | MIC - 25 µg/mL | [83]. |
| | Guignardone J (86), 6,8-Dihydroxy-5-methoxy-3-methyl-1H-isochromen-1-one (87), | Meroterpenes, Polyketides | | | *P. aeruginosa* and *S. aureus*, | MIC - 50 µg/mL | |
| | 3,4-dihydroxybenzoic acid (88) | Polyketides | | | *P. aeruginosa* and *S. aureus*, | MIC - 25 µg/mL | |
| 38. | Nigronaphthaphenyl (89) | Naphtho-phenyl ketone | *Nigrospora sphaerica* | *Bruguiera gymnorrhiza* collected at Attaragoda Wetland, Galle, Sri Lanka. | *S. aureus* (ATCC 43300) and MRSA (ATCC 33591), | MIC - 4 and 2 µg/mL | [84]. |
| | | | | | *P. aeruginosa* (ATCC 27853) | MIC - 2 µg/mL | |
| 39. | Daldinaphchromes A–C (90-92), | Polyketide | *Daldinia eschscholzii* HJX1P2 | Hainan Mangrove Conservation Area, Haikou | *S. aureus* (ATCC 29213) - | MIC - 74.4, 74.4, and 148.8 µM | [85]. |
| | 8-Ethyl-7-hydroxy-2,3-dimethyl-4-oxo-chromene-5-carboxylic acid (93) and 8-Methoxynaphthalene-1,7-diol (94) | Polyketide Methoxynaphthalenes | | | MRSA (ATCC 700699), MRSA (ATCC 43300) | MIC - 132.2 and 131.6 Mm MIC - 390.6 and 263.2 µM | |
| 40. | 8-O-Methylnodulisporin F (95) and Nodulisporin H (96) | Polyketides | *Daldinia eschscholtzii* HJ004, | *Bruguiera sexangula var. rhynchopetala* | *S. aureus* and MRSA, | MIC values range from 6.25 to 12.5 µg/mL. | [86]. |

| | | | | | | | |
|---|---|---|---|---|---|---|---|
| | | | | *collected in the South China Sea.* | | | |
| 41. | Parengyomarin A (97) and Parengyomarin B (98), Torrubiellin B (99) | Torrubiellin derivatives, Torrubiellin derivatives, Anthraquinone dimer | *Parengyodontium album* | *Avicennia marina* in Dong Zhai Gang Mangrove Garden, Hainan, China | *S. aureus* and MRSA, | MIC values range from 0.39 to 3.12 μM. | [87]. |
| 42. | Xylitol (100) | Sugar Alcohol | *Pestalotia* sp. | *Heritiera fomes collected from the Sundarbans* | MRSA strains (XU212, ATCC25923, SA1199B, EMRSA-15, and 340702) | MIC - 128, 128, 64, 64, and 128 μM, | [88]. |
| | Oxysporone (101) | Heterocyclic compound | | | MRSA strains (XU212, ATCC25923, SA1199B, EMRSA-15, and 340702) | MIC - 128, 64, 32, 32, and 64 μM | |
| 43. | (11S)-1,4,6-trihydroxy-7-(1-hydroxyethyl)-3-methoxyanthracene-9,10-dione (102), | Anthraquinone | *Fusarium* sp. J3-2, | Soil from Dongzhaigang Mangrove Natural Reserve, Hainan Province, China | *Staphylococcus aureus* ATCC 43300, ATCC 25923, ATCC 29213, *Enterococcus faecalis* ATCC 51299, and *E. faecium* ATCC 35667 | MIC values range from 25 to 50 μg/mL. | [89]. |
| | anhydrofusarubin (103), | Naphthoquinone | | | *E. faecalis* ATCC 51299 | MIC - 25 μg/mL | |

| | | | | | | | |
|---|---|---|---|---|---|---|---|
| | (3S)-3,8-dihydroxy-6,imethyl-α-tetralone (104) | Polyketide | *Cladosporium* sp. JJM22 | *Ceriops tagal* collected from the South China Sea | *S. aureus* and MRSA | Active at a concentration of 20 μM | [90]. |
| 44. | Cladocladosin A (105) | Macrolide | *Cladosporium cladosporioides MA-299* | *Bruguiera gymnorrhiza* collected on Hainan Island. | *Pseudomonas aeruginosa* | MIC - 4.0 μg/mL | [91]. |
| 45. | 5R-Hydroxyrecifeiolide (106) | Macrolides | *Cladosporium cladosporioides* MA-299 | *Bruguiera gymnorrhiza* collected from Hainan Island, China | *P. aeruginosa* | MIC - 32 μg/mL | [92]. |
| | ent-cladospolide F (107) | Polyketide | | | *S. aureus* | MIC - 8.0 μg/mL | |
| 46. | 1,1′-Dioxine-2,2′-dipropionic acid (108), 2-Methylacetate-3,5,6-trimethylpyrazine (109), Vermistatin (110), Citrinin H1 (111), Ladosporol E (112), Cladosporol C (113), and Cytochalasin D (114) | Pentenoic acid derivative, Pyrazine class. Funicone-like compound, Polyketide, Polyketide/terpenoid metabolite, Tetralins, Polyketide | *Cladosporium* sp. JS1-2 | *Ceriops tagal* collected from the South China Sea | *S. aureus* | MIC - 25.0, 12.5, 25, 6.25, 1.56, 6.25, and 25 μg/mL | [93]. |
| 47. | (2S)-2,3-Dihydro-5,6-dihydroxy-2-methyl-4H-1-benzopyran-4-one (115) (119), (2′R)-2-(2′-Hydroxypropyl)-4-methoxyl-1,3-benzenediol (116), and 4-Ethyl-3-hydroxy-6-propenyl-2H-pyran-2-one (117), | Polyketides | *Colletotrichum gloeosporioides* | *Ceriops tagal* collected from Dong Zhai Port, Hainan Province, China. | *S. aureus* | MIC - 12.5 μg/mL, | [94] |

| | | | | | | | |
|---|---|---|---|---|---|---|---|
| 48. | Dothiorelones K and M (118-119) | Cytosporone derivatives | *Dothiorella* sp. ML002 | *Xylocarpus granatum* from the South China Sea | *Staphylococcus aureus* ATCC 6538 | MIC - 50 µg/mL | [95]. |
| 49. | (+)-(R)-de-O-methyl-lasiodiplodin (120) | Benzenediol Lactone | *Lasiodiplodia theobromae* M4.2-2 | Mangrove sediment collected at Dongzhai Harbor, Hainan, China. | *S. aureus* ATCC 29213 and ATCC 700699 | MIC - 25 µg/mL | [96]. |
| 50. | 6-Hydroxy-astropaquinone B (121) and astropaquinone D (122), 3-O-methyl-9-O-methylfusarubin (123), | Naphthoquinone derivatives, Polyketide, naphthoquinone | *Fusarium napiforme* | *Rhizophora mucronata* collected in Takalar Regency, South Sulawesi Province, Indonesia | *S. aureus* NBRC 13276<br><br>*P. aeruginosa* ATCC 15442 | MIC - 6.3, 12.5, and 6.3 µg/mL<br><br>MIC - 6.3 µg/mL | [97]. |
| | astropaquinones B (124), and C (125), | Pyranonaphthoquinone | | | *S. aureus* YMF 3.17 | MIC - 20 and 10 µg/mL | |
| 51. | Dichlorodiaportintone (126), | Polyketides | *Ascomycota* sp. CYSK-4 | *Pluchea indica* collected from Shankou Mangrove Nature Reserve, Guangxi Province, China | *S. aureus* and *Klebsiella pneumoniae* | MIC - 50 µg/mL | [98]. |
| | Desmethyldichlorodiaportin (127) and Dichlorodiaportin (128) | Isocoumarin , Isocoumarin , | | | *S. aureus* and *Klebsiella pneumoniae* | MIC values range from 25 and 50 µg/mL | |

| | | | | | | | |
|---|---|---|---|---|---|---|---|
| 52. | Dalesconosides A (129) and C (130), (4R)-4,8-Dihydroxy-3-hydroxy-5-methoxy-1-naphthalenone (131), (4R)-O-Methylsclerone (132), (4R)-3,4-dihydro-4,5-Dihydroxynaphthalen-1(2H)-one (133), Fusaraisochromenone (134), 2-Acetyl-7-methoxybenzofuran (135), (3S)-3,8-Dihydroxy-6,7-dimethyl-α-tetralone (104), | Naphthalene derivatives, Naphthalene derivatives, Polyketide-derived *N*aphthalenone. Naphthoquinone derivative, Benzofuran and furan<br><br>Polyketide | *Daldinia eschscholzii* MCZ-18 | *Ceriops tagal* collected from Dong Zhai Gang Mangrove Garden, Hainan Island, China. | *P. aeruginosa*, *Enterococcus faecalis*, and MRSA, | MIC values ranging from 6.25 to 25 µg/mL, | [99]. |
| 53. | Colletotric B (136) | Polyketide | *Phoma sp. SYSU-SK-7* | *Kandelia candel* collected from Shankou Mangrove Nature Reserve, Guangxi Province, China | MRSA and *P. aeruginosa* | MIC - 3.36 and 1.67 µg/mL | |
| | 3-Hydroxy-5-methoxy-2,4,6-trimethylbenzoic acid (137) | Polyketide | | | | | |
| | Colletotric A (138) | Polyketide | | | MRSA and *P. aeruginosa* | MIC - 6.28 and 3.37 µg/mL. | |
| | Orsellinic acid (139) | Polyketide | | | MRSA and *P. aeruginosa* | MIC - 8.40 and 2.10 µg/mL. | |
